# Microbial diversity drives multifunctionality in terrestrial ecosystems

**DOI:** 10.1038/ncomms10541

**Published:** 2016-01-28

**Authors:** Manuel Delgado-Baquerizo, Fernando T. Maestre, Peter B. Reich, Thomas C. Jeffries, Juan J. Gaitan, Daniel Encinar, Miguel Berdugo, Colin D. Campbell, Brajesh K. Singh

**Affiliations:** 1 Hawkesbury Institute for the Environment, Western Sydney University, Penrith, New South Wales 2751, Australia; 2 Área de Biodiversidad y Conservación, Departamento de Biología y Geología, Física y Química Inorgánica, Escuela Superior de Ciencias Experimentales y Tecnología, Universidad Rey Juan Carlos, Calle Tulipán Sin Número, Móstoles 28933, Spain; 3 Department of Forest Resources, University of Minnesota, St Paul, Minnesota 55108, USA; 4 Instituto de Suelos, CIRN, INTA, Nicolas Repetto y de los Reseros Sin Número, Hurlingham, Buenos Aires 1686, Argentina; 5 The James Hutton Institute, Craigiebuckler, Aberdeen AB15 8QH, UK; 6 Global Centre for Land-Based Innovation, Western Sydney University, Penrith South DC, New South Wales 2751, Australia

## Abstract

Despite the importance of microbial communities for ecosystem services and human welfare, the relationship between microbial diversity and multiple ecosystem functions and services (that is, multifunctionality) at the global scale has yet to be evaluated. Here we use two independent, large-scale databases with contrasting geographic coverage (from 78 global drylands and from 179 locations across Scotland, respectively), and report that soil microbial diversity positively relates to multifunctionality in terrestrial ecosystems. The direct positive effects of microbial diversity were maintained even when accounting simultaneously for multiple multifunctionality drivers (climate, soil abiotic factors and spatial predictors). Our findings provide empirical evidence that any loss in microbial diversity will likely reduce multifunctionality, negatively impacting the provision of services such as climate regulation, soil fertility and food and fibre production by terrestrial ecosystems.

## Abstract (web summary)

The role of microbial diversity in ecosystems is less well understood than, for example, that of plant diversity. Analysing two independent data sets at a global and regional scale, Delgado-Baquerizo *et al*. show positive effects of soil diversity on multiple terrestrial ecosystem functions.

## Graphical abstract

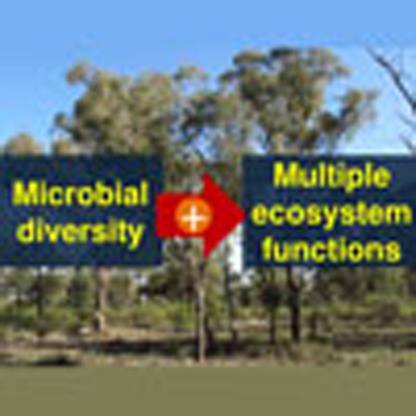

A large body of research conducted during the past two decades indicates that ecosystem functioning is positively related to plant diversity^1^,^2^,^3^,^4^. Unlike plants, we have limited knowledge of the relationship between microbial diversity and ecosystem functioning, particularly in terrestrial environments^5^,^6^. Microbial communities play key roles in maintaining multiple ecosystem functions and services simultaneously (‘multifunctionality' hereafter), including nutrient cycling, primary production, litter decomposition and climate regulation^7^,^8^,^9^,^10^. Experiments carried out under controlled conditions^8^,^11^,^12^,^13^ suggest that the diversity of soil organisms can promote multifunctionality. However, none of these studies have explicitly addressed the relationship between soil microbial diversity and multifunctionality on the global scale.

Of the various ecosystem processes on Earth, plant productivity and nutrient cycling are among those most important for supporting human welfare^9^,^14^. Because of the continuous global population growth^15^, substantial increases in plant production and land use intensification will be required to support future demand for food and fibre^14^. Understanding the factors controlling the multiple functions linked to plant production and nutrient cycling under a changing environment is, thus, critical to preserve and manage natural and human-dominated ecosystems. We posit that soil microbial diversity plays a key role in maintaining ecosystem multifunctionality by supporting processes such as litter decomposition and organic matter mineralization^3^,^7^,^16^,^17^, which allow transfer of matter and energy between above- and belowground communities^12^,^16^,^17^,^18^,^19^. There is a growing body of experimental and observational studies providing evidence that the relationship between biodiversity (that is, microbes and plants) and ecosystem functioning is more linear than saturating^6^,^20^,^21^. Thus, any loss in microbial diversity as a consequence of global environmental changes such as land use, nitrogen enrichment and climate change^3^,^9^,^10^,^14^,^22^,^23^ would likely alter the capacity of microbes to sustain multiple above- and belowground ecosystem functions. However, we lack empirical evidence on the relationships between microbial diversity and multifunctionality in terrestrial ecosystems, and few studies have addressed the relative importance of this diversity versus other drivers of ecosystem functioning, such as soil abiotic properties, climate and plant species richness^8^,^18^. This hampers our ability to predict changes in multifunctionality under ongoing global environmental change, and to formulate sustainable management and conservation policies^10^.

Here, we hypothesize that microbial diversity: (i) promotes multifunctionality in terrestrial ecosystems; and (ii) is as important as variables such as soil pH, climate and spatial predictors, latitude and altitude as drivers of variation in multifunctionality. We tested these hypotheses using data from two large-scale surveys, a global study including 78 drylands from all continents except Antarctica (‘Drylands' hereafter)^24^,^25^ and a national soil survey including 179 locations in Scotland (‘Scotland' hereafter)^26^. The Drylands data set include diverse ecosystem types (grasslands, mixed grassland/woodland and woodlands), and provides a wide range of environmental conditions typically found in drylands worldwide. Similarly, the Scotland data set includes six ecosystem types (bog, moorland, semi-natural grassland, forest, arable and improved grassland) covering the whole of Scotland, and is representative of many soil types and land uses found in northern temperate regions. Our intention is not to merge both data sets, which indeed have some differences in sampling design and experimental methods, but to test our hypotheses using two independent and large-scale data sets from ecosystems widely differing in their vegetation, climatic and soil attributes^24^,^25^,^26^.

We found that soil microbial diversity is positively related to multifunctionality in both the Drylands and the Scotland data sets. The positive effects of microbial diversity on multifunctionality were maintained even when accounting simultaneously for multiple climatic, abiotic and spatial predictors of multifunctionality. Our study provides empirical evidence that microbial diversity positively relates to multifunctionality in terrestrial ecosystems on the global scale; and further suggests that any loss in microbial diversity will likely reduce the rates at which multiple ecosystem functions and services are being maintained in terrestrial ecosystems.

## Results and Discussion

### Microbial diversity and ecosystem multifunctionality

**Figure 1:**
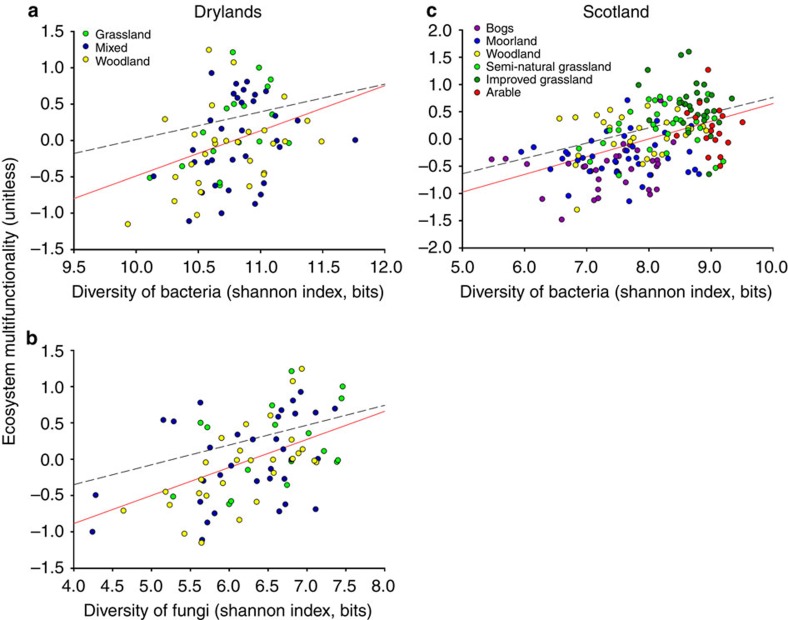
Relationships between microbial diversity and ecosystem multifunctionality. Results are shown for the Drylands (bacteria (**a**) and fungi (**b**)) and Scotland (bacteria (**c**)) data sets. The solid and dashed lines represent the fitted ordinary least squares (OLS) and simultaneous autoregression (SAR) models, respectively. Results of regressions are as follows: (**a**) OLS, *R*^2^=0.118, *P*=0.012, AICc=133.463; SAR, *R*^2^=0.101, *P*=0.005, AICc=135.013; (**b**) OLS, *R*^2^=0.235, *P*=0.002, AICc=122.399; SAR, *R*^2^=0.215, *P*<0.001, AICc=124.433 (**c**) OLS, *R*^2^=0.226, *P*<0.001, AICc=265.539; SAR, R^2^=0.222, *P*<0.001, AICc=266.574.

A total of 166,244/24,249 (bacteria/fungi) and 49,102 (bacteria) operational taxonomic units (OTUs) were found in the Drylands and Scotland data sets, respectively (see Supplementary Fig. 1 for rarefaction curves and Supplementary Figs 2 and 3 for the dominant taxa found). We first explored the relationship between microbial diversity, estimated with the Shannon index (ref. 27), and multifunctionality, evaluated using the standardized average of six variables that were available for the two data sets: potential net nitrogen (N) mineralization, nitrate, ammonium, DNA concentration, available phosphorus (P) and plant productivity (see Methods). Soil microbial diversity positively relates to multifunctionality in both data sets (Fig. 1). These results were maintained when controlling for the spatial structure of the data by using spatial autoregressive analyses^25^,^28^ (Fig. 1). We also found positive relationships between soil microbial diversity and most of the individual functions measured, as well as between this diversity and most of the possible combinations among functions (Supplementary Table 1). Our multifunctionality index was also strongly related, for each data set, to an extended version of this index including 8 and 17 soil functions that were unique to the Scotland and Drylands data sets, respectively (Supplementary Figs 4 and 5). Further analyses provided evidence that Shannon diversity was positively and strongly related to biodiversity components such as phylogenetic diversity and species richness (Supplementary Figs 6–9). Phylogenetical diversity and species richness were also highly and positively related to multifunctionality (Supplementary Figs 6–9). Finally, our results were robust to the approach used to quantify multifunctionality: single functions (Supplementary Table 1), averaging multifunctionality (Fig. 1) and multiple-threshold multifunctionality (Supplementary Figs 10 and 11). The multiple-threshold approach provided additional evidence that the effect of microbial diversity in the number of functions surpassing different thresholds of functionality is mainly positive and significant (Supplementary Figs 10 and 11). Also, the maximum number of functions maximized is the same than the number of functions measured (six, see Supplementary Fig. 10), which indicates that there are no trades-offs between the functions evaluated in our study. Moreover, the multiple-threshold approach indicated that the effect of diversity over multifunctionality is moderate-high in the Drylands data set and high in the Scotland data set (see Supplementary Figs 10 and 11). In particular, these results indicate that microbial diversity in Drylands has a significant effect on the ability of the system to provide more functions working at moderate to high performance levels (peaks ∼40 and 60% in Supplementary Fig. 11a,b respectively), whereas in Scotland this is expanded to functions working at really high performance levels (peak ∼75% in Supplementary Fig. 11c). Albeit our results are correlative in nature, and hence cannot be taken as a definitive proof of causation, they agree with those from theoretical and experimental studies showing a positive relationships between overall soil diversity and multiple soil functions, such as those used here^8^,^12^,^13^,^16^,^29^,^30^. Moreover, a recent field observational study has also found positive relationships between bacterial diversity and multifunctionality in the Chinese Tibetan Plateau^18^. Our results provide, to our knowledge, the first empirical evidence showing that microbial diversity positively relates to multifunctionality in terrestrial ecosystems on the global scale. Consequently, our results support the hypothesis that microbial diversity can be critical to maintain multifunctionality^8^,^18^, suggesting that losses of microbial diversity will likely reduce the ability of terrestrial ecosystems to provide critical ecosystem services.

### Accounting for multiple multifunctionality drivers

**Figure 2:**
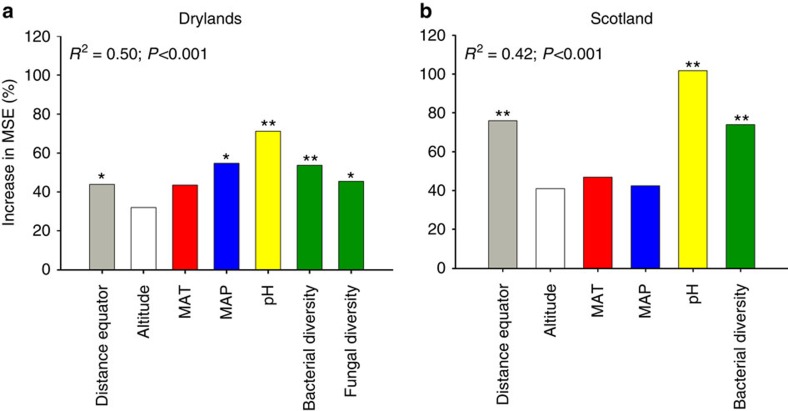
Main predictors of ecosystem multifunctionality. The figure shows the Random Forest mean predictor importance (% of increase of MSE) of environmental drivers and microbial diversity (Shannon index, bits) on ecosystem multifunctionality for the Drylands (**a**) and Scotland (**b**) data sets. Significance levels of each predictor are as follows: *P<0.05 and **P<0.01. MAT, mean annual temperature; MAP, mean annual precipitation.

**Figure 3:**
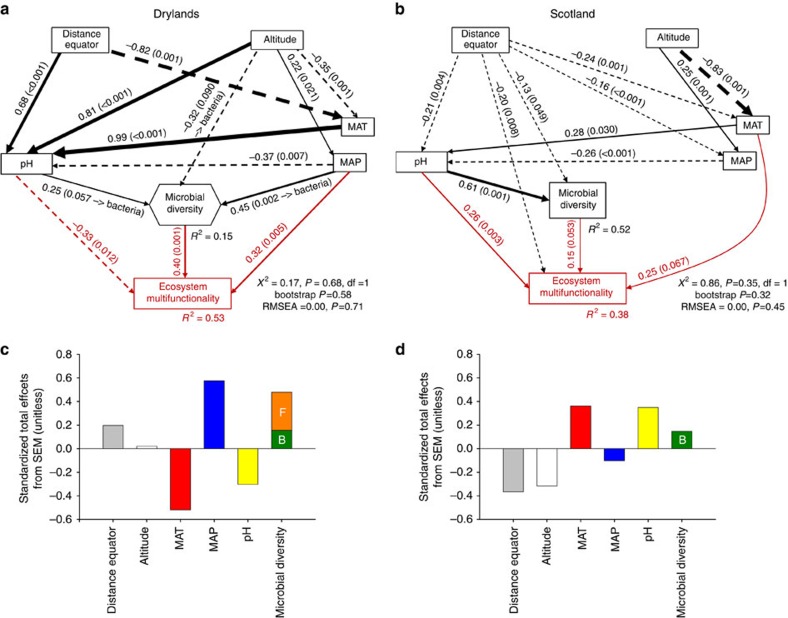
Direct and indirect effects of space, climate, soil pH and microbial diversity on ecosystem multifunctionality. Structural equation models are shown for the Drylands (**a**,**c**) and Scotland (**b**,**d**) data sets. Numbers adjacent to arrows are indicative of the effect-size (bootstrap *P* value) of the relationship. Continuous and dashed arrows indicate positive and negative relationships, respectively. Spatial effects have no +/− sign. The width of arrows is proportional to the strength of path coefficients. *R*^2^ denotes the proportion of variance explained. (**c**,**d**) Standardized total effects (direct plus indirect effects) derived from the structural equation models depicted above. Capital letters B and F within standardized total effects bars indicate the effects of bacterial and fungal diversity, respectively, in the Drylands data set. MAT, mean annual temperature; MAP, mean annual precipitation.

We used Random Forest modelling^31^ to identify the most important predictors (distance from equator, altitude, mean annual temperature (MAT), mean annual precipitation (MAP), soil pH and microbial diversity) of multifunctionality; and structural equation modelling (SEM) (ref. 32) to test whether the relationship between microbial diversity and multifunctionality is maintained when accounting for multiple multifunctionality drivers simultaneously (see *a priori* model in Supplementary Fig. 12). Our Random Forest models indicate that microbial diversity was as important as or more important than other multifunctionality predictors (Fig. 2). Indeed, microbial diversity was more important than MAT and altitude in the two data sets, and than MAP in the Scotland data set (Fig. 2b). Similar results were found after including ecosystem type as a predictor in these analyses (Supplementary Fig. 13; see Supplementary Figs 14 and 15 for values of the functions measured across ecosystem types). The role of distance from equator, altitude, climate and soil pH as predictors of multifunctionality is well known^17^,^25^. Most relevant to the topic of this study, we found that microbial diversity was a major predictor of multifunctionality in the two data sets used, even after accounting for the simultaneous direct and indirect effects of these variables (Fig. 2a,b). Our SEMs explained 53 and 38% of the variance found in the ecosystem multifunctionality of the Drylands and Scotland data sets, respectively (Fig. 3a,b). In both cases we found a direct positive effect of microbial diversity on multifunctionality (Fig. 3).

In the Drylands data set, fungal diversity showed a slightly higher total positive effect than bacterial diversity on multifunctionality. Fungi are known to be more tolerant of desiccation than bacteria^33^, and thus fungal diversity may have a predominant effect on multifunctionality in drylands, where soils remain under dry conditions during most of the year^34^. Not surprisingly, the effects of climate and soil pH on multifunctionality followed opposite trends in the Drylands and Scotland data sets, as indicated by the standardized total effects from SEM (Fig. 3c,d). The biological activity and productivity of drylands are well known to be limited by rainfall, rather than by temperature (except in cold deserts)^34^; consistent with this, MAP and multifunctionality were positively related in our data set. Contrarily, temperature, but not MAP, is known to limit ecosystem functioning in mesic cold temperate ecosystems such as those from Scotland^35^. Similarly, soil pH is often basic in drylands (for example, because of carbonate accumulation) and acid in cold temperate ecosystems (for example, due to organic matter accumulation)^35^. Therefore, assuming microbes are adapted to the typical pH of their habitats, soil pH influences multifunctionality in a distinct manner in the two data sets studied. Despite these contrasting effects, the positive direct and total effects of microbial diversity on multifunctionality were always maintained, and were robust to the analytical methods used here (linear regression, random forest and SEM). Collectively, these results demonstrate that microbial diversity plays critical roles supporting ecosystem functioning in terrestrial ecosystems.

**Figure 4:**
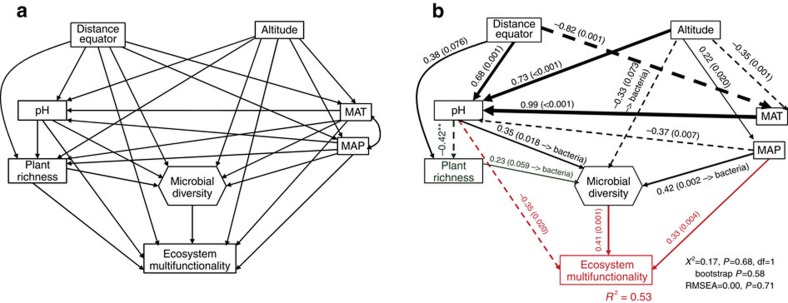
Direct and indirect effects of space, climate, soil pH, plant richness and microbial diversity on ecosystem multifunctionality in global drylands. *A priori* (**a**) and final (**b**) structural equation models including plant richness as a predictor of ecosystem multifunctionality are shown. Numbers adjacent to arrows indicate the effect-size of the relationship. Continuous and dashed arrows indicate positive and negative relationships, respectively. The width of arrows is proportional to the strength of path coefficients. *R*^2^ denotes the proportion of variance explained.

Microbial diversity can support multifunctionality in a wide variety of ways. For example, microbial communities carry out critical ecological processes such as decomposition and nutrient cycling^3^,^7^,^16^,^17^, and, thus, can support the fundamental mechanisms linking aboveground and belowground communities in terrestrial ecosystems^3^,^7^,^16^,^17^. Supporting this idea, we found that the previously reported positive effects of plant richness on multifunctionality in the Drylands data set^25^ may be indirect, and result from positive effects of plant richness on microbial diversity (Fig. 4). This result is consistent with studies showing that microbial driven enhancement of soil nitrogen cycling typically associated to high plant diversity levels stimulates productivity^21^. Highly diverse plant communities may promote the diversity of soil microbes by supporting a wide variety of litter qualities^16^. A greater microbial diversity can enhance the rapid break down of litter derived from aboveground communities, increasing soil organic matter content and fostering the activity of soil microbial communities^12^,^17^. Similarly, organic matter needs to be degraded from complex and recalcitrant polymers into simpler and more labile monomers, a process requiring the cooperation of a large and diverse group of microorganisms^3^,^7^,^16^,^17^. During this process, soil nutrients are released by microbes and are again available for aboveground communities, supporting important ecosystem services such as food and fibre production^17^. Thus, though largely overlooked, microbial diversity supports multifunctionality by altering nutrient supply and the distribution of resources^3^,^7^,^16^,^17^, enabling high rates of material processing in terrestrial ecosystems.

## Conclusions

Altogether, our findings provide strong empirical evidence that, similarly to what has been found with plants and animals^3^,^4^, microbial diversity is critical for maintaining the multifunctionality of terrestrial ecosystems. The message for scientists, policy makers, educators and organizations involved in understanding biodiversity patterns, microorganisms and ecosystem functioning is clear: losses in microbial diversity derived from human activities and climate change will reduce the rates at which multiple ecosystem functions and services are being maintained. By providing evidence for the relationship between microbial diversity and multifunctionality, our findings advance key ecological topics such as biodiversity–ecosystem functioning relationships in microbial communities. These findings emphasize the need to develop approaches and policies to protect soil microbial diversity from global environmental drivers such as land use, nitrogen enrichment and climate change, so that the multifunctionality of terrestrial ecosystems is to be preserved for future generations.

## Methods

### Study sites and data collection

*Drylands*. We used a subset of 224 sites from the global dryland network presented in Maestre *et al*.^25^ This network targets dryland ecosystems, defined as regions with an aridity index (AI=precipitation/potential evapotranspiration) between 0.05 and 0.65 (ref. 36). Field data were collected between 2006 and 2012 from 78 sites located in 12 countries from all continents except Antarctica according to a standardized sampling protocol (ref. 25). The choice to analyse a subset of sites was largely logistical, as we were only able to obtain frozen soils from a subset (see ref. 24) of the 224 original sites surveyed in Maestre *et al*. (25). At each site, a 30 m × 30 m plot was established under the most representative vegetation. A composite sample (that is, from five soil samples; 0–7.5 cm depth) was randomly taken under the canopy of the dominant perennial plant species and in open areas devoid of perennial vegetation. After field collection, moist soil samples were taken to the laboratory and sieved (<2 mm). Each sample was separated into two portions. The first portion was air dried for chemical and functionality analysis. The second portion was stored at −20 °C until DNA extractions could be performed. To avoid problems associated with the use of multiple laboratories when analysing the soils from different sites, and to facilitate the comparison of results between them, dried and frozen soil samples from all the countries were shipped to Spain (laboratories of Pablo de Olavide University and Rey Juan Carlos University) for analyses.

*Scotland*. We used data from the soil sampling conducted during 2006–2009 as part of the Second National Soils Inventory of Scotland^26^. Field data were collected from 179 sites across Scotland, using a 20 × 20 km sampling grid. Each site included a central pit where a soil sample was collected from the uppermost horizon of soil under the most representative plant community^26^. Field moist soils were sieved to <4 mm and visible pieces of plant material, and soil animals were removed before use. The details and protocols for soil sampling and profile description are given in Yao *et al*.^26^ Each sample was separated into three portions. The first portion was air dried for chemical analysis (that is, pH). The second portion was stored at 4 °C for the assessment of soil functions. The third portion was stored at −20 °C until DNA extractions could be performed. To avoid problems associated with the use of multiple laboratories when analysing the soils from different sites, all chemical and soil functionality analyses were conducted in Scotland (James Hutton Institute; chemical and soil functionality analysis).

*Abiotic variables*. Soil pH was measured in all the soil samples with a pH meter, in a soil and water suspension. In addition, for each plot, we collected data on MAP and MAT and altitude from http://www.worldclim.org/37.

### Assessing microbial diversity

*Drylands (fungi and bacteria)*. DNA was extracted from 0.5 g of defrosted soil samples using the Powersoil DNA Isolation Kit (Mo Bio Laboratories, Carlsbad, CA, USA) according to the instructions provided by the manufacturer. The extracted DNA was frozen and shipped to the Next-Generation Sequencing Facility of the Western Sydney University, where they were defrosted and analysed using the Illumina MiSeq platform and 341F/805R (bacteria) and FITS7/ITS4 (fungi) primer sets^38^,^39^. Initial sequence processing and diversity analyses for both bacterial 16S rDNA and fungal ITS genes were conducted using the QIIME package^40^. Initially, low-quality regions (Q<20) were trimmed from the 5′ end of sequences and paired ends were joined with FLASH for 16S rDNA sequences and Fastq-join^41^ for ITS reads. Sequences were de-multiplexed and a further round of quality control conducted to remove sequences containing ambiguous bases (N), and reads containing bases with a quality score below 25. Chimeric 16S rDNA sequences were detected using the UCHIME algorithm from the USEARCH package^42^ implemented within VSEARCH (https://github.com/torognes/vsearch). The RDP training data set V9 (ref. 43) was used as a reference for chimaera detection as recommended by the UCHIIME documentation. *De novo* (abundance based) chimaera detection was used for ITS data using USEARCH (ref. 42). The remaining high-quality chimaera-free sequences were used for downstream analysis. A total of 15489774 and 19290226 sequences were obtained for bacteria and fungi, respectively. OTUs were defined as clusters of 97% sequence similarity using UCLUST (ref. 42). Taxonomy was assigned using UCLUST (ref. 42) against the Greengenes database version 13_8 (refs 44, 45) for 16S rDNA OTUs. For fungal ITS sequences, taxonomy was assigned using BLAST (refs 46, 47) against the UNITE database V6.9.7. (E<10^−5^). The resultant OTU abundance tables for both primer sets were filtered to remove singletons and rarefied to an even number of sequences per samples to ensure equal sampling depth (25113 and 23588 for 16S rDNA and ITS, respectively). Our Drylands data set included a total of 166,244 and 24,249 OTUs for the 16S and ITS genes. The Shannon diversity index was calculated on these rarefied OTU tables using QIIME (ref. 42); we selected this metric for our study because it provides a robust and informative estimation of taxonomic diversity for microbial communities^27^.

*Scotland (bacteria)*. DNA was extracted from 0.5 g defrosted soil samples of a unique soil sample per site (sample collected under the central pit) using the FastDNA SPIN kit for soil (Bio101, Vista, CA), according to the manufacturer's protocol. The extracted DNA was frozen and shipped to the Next-Generation Sequencing Facility of the Western Sydney University, where they were defrosted and analysed using amplicon 454 pyrosequencing and 341F/806R (bacteria) primer set^38^. Pyrosequencing of 16S rRNA gene was performed on a Roche GS FLX System and Titanium kit. Barcode, linker primer and reverse primer sequences were removed from the raw sequence reads using the ‘split_libraries.py' script while setting minimum sequence length of 200 and minimum quality score of 20. ‘Acacia' tool was used with default options to remove pyrosequencing noise^48^. Potential chimaeras were removed using the UCHIME chimaera detection (*de novo* mode) utility of the USEARCH v6.0.307 tool (ref. 42). Similar sequences were binned into OTUs using ‘UCLUST' method (minimum pairwise identity of 97%). Our Scotland data set included a total of 49,102 OTUs for the 16S gene. We calculated the Shannon diversity index using the ‘Quantitative Insights Into Microbial Ecology' (QIIME v 1.6.0) software package^42^ rarefacted at 1,128 sequences per sample.

*Phylogenetic diversity (Both drylands and Scotland data sets)*. Biodiversity involves multiple components including, but not limited to, species richness, evenness, composition, phylogenetic diversity and functional diversity^6^,^49^. While the Shannon index encompasses both species richness and evenness, and has been widely used to characterize the diversity of microbial communities^18^,^29^,^50^ recent studies have emphasized the importance of phylogenetic diversity as an important driver of ecosystem functioning^51^. Thus, we calculated the bacterial phylogenetic diversity for both the Dryland and Scotland data sets. Representative sequences from each OTU were aligned using PyNAST (ref. 40) and filtered to remove uninformative regions. A phylogenetic tree was then constructed using FastTree^52^ and the phylogenetic diversity was calculated from this tree using Faith's metric^53^, which is based on the total branch length of the tree. We did not calculate fungal phylogenetic diversity as unifrac analyses are not recommended for the ITS gene. This is because it is not possible to generate an accurate alignment for ITS because of the high variability of fragment size for this particular gene.

### Assessing ecosystem multifunctionality

*Both Drylands and Scotland*. For this study, we used six variables that were available for the two data sets: potential net nitrogen (N) mineralization, nitrate, ammonium, DNA concentration, available phosphorus (P) and plant productivity. Overall, these variables constitute good proxies of processes driving nutrient cycling, biological productivity, and the build-up of nutrient pools^25^,^54^. In particular, N and P are the nutrients that most frequently limit the primary production in terrestrial ecosystems^35^. For example, ammonium and nitrate are important N sources for both microorganisms and plants^35^. In addition, potential net N mineralization is a key processes within the N cycle transforming organic into inorganic N. Inorganic P is the main P source for plants and microorganisms^35^, and its availability is linked to the desorption and dissolution (for example, through oxalate exudates) of P from soil minerals, and to a lesser extent, to the decomposition of organic matter^35^. In addition, DNA concentration has been recently used as a proxy of surface soil biomass^55^,^56^. In the Scotland data set, this variable is strongly related to the glucose substrate-induced respiration (Spearman's *ρ*=0.70; *P*<0.001), a common proxy of soil microbial biomass^57^. In addition, as a molecule rich in N and P, DNA could be an important source of microbial nutrition^58^. Finally, plant productivity is a key ecosystem process that sustains human welfare, support belowground ecosystem functionality^17^,^59^, and plays major roles in the global carbon cycle^17^,^59^.

Extractable ammonium and nitrate were obtained from K_2_SO_4_ and KCl extracts in the Drylands and Scotland data sets, respectively. The potential net N mineralization rate was estimated as the difference between initial and final inorganic N (sum of ammonium and nitrate) before and after incubation under potential conditions^60^ in both data sets. Soil phosphorus was estimated from sodium bicarbonate^61^ and acid ammonium oxalate^62^ extracts in the Drylands and Scotland data sets, respectively. In both cases, the concentration of DNA was estimated with a Nanodrop 2000 UV–vis spectrophotometer (Wilmington, USA) after DNA extraction as described above. Finally, we used the Normalized Difference Vegetation Index (NDVI) as our proxy of plant productivity^59^. These data were obtained from the Moderate Resolution Imaging Spectroradiometer (MODIS) aboard NASA's Terra satellites (http://daac.ornl.gov/index.shtml). NDVI provides a global measure of the ‘greenness' of vegetation across Earth's landscapes for a given composite period, and thus acts as a proxy of photosynthetic activity and large-scale vegetation distribution^59^. Here, we used averaged values obtained from NDVI values for the months before, during and after sampling at each of the surveyed plots. This index was calculated in the same way for both the Drylands and Scotland data sets.

Because of the huge differences in bulk density among soil samples in the Scotland data set (0.06–1.35 g cm^−3^), all the soil functions were corrected to account for the different bulk density values observed in each of the surveyed plots. Bulk density information was not available for the Drylands data set, but we do not expect vast differences in bulk density among dryland ecosystems due to the mineral nature of their soils. In fact, in a subset of our data set where bulk density was available, multifunctionality estimates corrected by bulk density were highly correlated with those non-corrected (ρ=0.763; *P*<0.001; *n*=25). In the Drylands data set, samples were collected in open areas and under the main vegetation; thus, all the soil variables in this data set were averaged to obtain site-level estimates by using the mean values observed in bare ground and vegetated areas, weighted by their respective cover at each site^25^.

### Assessing multifunctionality

Multifunctionality is a human construct rather than a single measurable process, and involves quantifying the provision of multiple ecosystem processes and services simultaneously^63^,^64^. These include, among other, nutrient cycling (for example, nutrient availability, mineralization), primary production (for example, net primary productivity) and organic matter decomposition (for example, lignin degradation). To obtain a quantitative multifunctionality index for each site, we first normalized (log-transform when needed) and standardized each of the six functions measured (ammonium, nitrate, potential net N mineralization, soil phosphorus, DNA content and plant productivity) using the *Z*-score transformation. These standardized ecosystem functions were then averaged to obtain a multifunctionality index^25^. This index is widely used in the multifunctionality literature^4^,^8^,^25^,^63^,^64^, and provides a straightforward and easy-to-interpret measure of the ability of different communities to sustain multiple functions simultaneously^4^,^8^,^25^. The multifunctionality index was independently obtained for the Drylands and Scotland data sets. While we calculated our multifunctionality index based on the six functions that were available for both Drylands and Scotland data sets to facilitate the comparison and generalization of our results, another 11 and nine functions were available for each of these data sets, respectively. To further test whether the functions used to estimate multifunctionality could be biasing our results, we recalculated our averaging multifunctionality index including all the available functions available per data set (eight and 17 for the Scotland and Drylands data sets, respectively). These extra functions included glucose substrate-induced respiration^57^ and basal respiration^57^ (corrected by bulk density) in Scotland, and activity of phosphatase and β-glucosidase, dissolved organic N, proteins, aminoacids, phenols, aromatic compounds, hexoses, pentoses, HCl-P and potential N transformation rate in the Drylands data set^25^.

Multifunctionality averaging approaches such as the index used in this manuscript do not take into account the number of functions with high performance. This entails some problems because unevenly strong functions may bias this index to high multifunctionality performance when actually only few functions maximize (it might be necessary that different functions maximize at the same time to avoid potential limiting factors in the system). Also it does not allow to see potential trade-offs between functions, which might maximize when others minimize. To solve this problem, a threshold approach was used^63^. In this technique, every function is standardized using the maximum of its value within the data set. We transformed every observation of every function into a percentage of the maximum performance of each function. To control for potential artefacts derived from the fact that the maximum value is necessarily one only measure, we used as maximum value the average of the top 5% of all plots value. We aimed to evaluate the relationship between diversity and the number of functions which perform higher than a given threshold. Since the choice of a threshold for multiple functions is arbitrary, Byrnes *et al*.^63^ developed a method that basically performs regressions between the number of functions surpassing a threshold and the diversity throughout thresholds from 0 to 99%. Each threshold represents a level of functional performance and the regressions indicate whether diversity is able to increment the number of functions working beyond that level of performance.

We plotted the resulting regressions in Supplementary Fig. 10 (one colour per threshold). To evaluate the significance of these regressions we plotted the effect (slope of regression) of diversity versus number of functions along different thresholds with their confidence interval at 95% in Supplementary Fig. 11. These analyses were conducted using Matlab v.7.0 (The MathWorks, Inc., Natick, Massachusetts, United States).

### Statistical analyses

We conducted a classification Random Forest analysis^31^ to identify which were the main predictors of multifunctionality among the following variables: distance from equator (absolute latitude), altitude, MAT, MAP, soil pH and microbial diversity (bacteria and fungi for drylands and bacteria for Scotland). Although distance from equator and altitude cannot be considered true causal drivers of ecosystem attributes, they are often considered as surrogates of multiple, and unmeasured, drivers of ecosystem functioning and biodiversity^65^. In addition, we repeat these analyses including ecosystem type (bog, moorland, semi-natural grassland, forest, arable and improved grassland for Scotland data set; and grassland, mixed and woodland for the Dryland data set) as a predictor in our analyses. Random Forest is a novel machine-learning algorithm that extends standard classification and regression tree (CART) methods by creating a collection of classification trees with binary divisions^66^. Unlike traditional CART analyses, the fit of each tree is assessed using randomly selected cases (1/3 of the data), which are withheld during its construction (out-of-bag or OOB cases). The importance of each predictor variable is determined by evaluating the decrease in prediction accuracy (that is, increase in the mean square error (MSE) between observations and OOB predictions) when the data for that predictor is randomly permuted. This decrease is averaged over all trees to produce the final measure of importance^29^. This accuracy importance measure was computed for each tree and averaged over the forest (5,000 trees). These analyses were conducted using the randomForest package^67^ of the R statistical software, version 3.0.2 (http://cran.r-project.org/). The significance of the model and the cross-validated *R*^2^ were assessed with 5,000 permutations of the response variable (ecosystem multifunctionality) using the A3 R package for R (ref. 68). Similarly, the significance of the importance of each predictor on multifunctionality was assessed by using the rfPermute package for R^69^. These analyses were independently done for the Drylands and Scotland data sets.

We used SEM (ref. 32) to evaluate the direct and indirect relationships between distance from equator (absolute latitude), altitude, MAT, MAP, soil pH, microbial diversity (bacteria and fungi for Drylands and bacteria for Scotland) and multifunctionality. The first step in SEM requires establishing an *a priori* model based on the known effects and relationships among the drivers of macro and microorganisms diversity (Supplementary Fig. 12). Some data manipulation was required before modelling. We examined the distributions of all of our endogenous variables, and tested their normality. Altitude (Drylands) was log-transformed to improve normality. Similarly, soil pH (Drylands), distance from equator (Drylands) and MAT (both data sets) were square-transformed. In addition, the diversity of fungi and bacteria were included as a composite variable (microbial diversity) in the Drylands data set. The use of composite variables does not alter the underlying SEM model, but collapses the effects of multiple conceptually-related variables into a single-composite effect, aiding interpretation of model results^32^. With a good model fit (see below), we were free to interpret the path coefficients of the model and their associated *P* values. A path coefficient is analogous to the partial correlation coefficient, and describes the strength and sign of the relationship between two variables^32^. Since some of the variables introduced were not normally distributed, the probability that a path coefficient differs from zero was tested using bootstrap^34^. Bootstrapping is preferred to the classical maximum-likelihood estimation in these cases because in bootstrapping probability assessments are not based on the assumption that the data match a particular theoretical distribution. Thus, data are randomly sampled with replacement in order to arrive at estimates of s.e.m. that are empirically associated with the distribution of the data found in the samples^32^. When these data manipulations were completed, we parameterized our model using our data set and tested its overall goodness of fit. There is no single universally accepted test of overall goodness of fit for SEM, applicable in all situations regardless of sample size or data distribution. Here we used the *χ*2-test (χ^2^; the model has a good fit when χ^2^ is low (∼≤2) and *P* is high (traditionally >0.05)) (ref. 70) and the root MSE of approximation (RMSEA; the model has a good fit when RMSEA is low (∼≤0.05) and P is high (traditionally>0.05)) (ref. 70). In addition, and because some variables were not normal, we confirmed the fit of the model using the Bollen-Stine bootstrap test (the model has a good fit when the *P* value is high (traditionally>0.10)) (ref. 70). Furthermore, we calculated the standardized total effects of distance from equator (absolute latitude), altitude, MAT, MAP, soil pH and microbial diversity (bacteria and fungi for Drylands and bacteria for Scotland) on multifunctionality. The net influence that one variable has upon another is calculated by summing all direct and indirect pathways between the two variables. If the model fits the data well, the total effect should approximately be the bivariate correlation coefficient for that pair of variables^32^. All the SEM analyses were conducted using AMOS 20.0 (AMOS IBM, USA). All these analyses were done independently for the Drylands and Scotland data sets.

It is important to notice that we used Random Forest modelling to identify the most important predictors of multifunctionality; and SEM (ref. 32) to test whether the relationship between microbial diversity and multifunctionality is maintained when accounting for multiple multifunctionality predictors simultaneously. Both approaches provide complementary insights on the patterns that drive multifunctionality at a large scale. For instance, Random Forest does not rely on *a priori* hypotheses, which need to be established before SEM analyses, hence its results are not biased by our previous knowledge. However, the use of SEM is particularly useful in large scale correlative studies^32^, as it allows us to partition causal influences among multiple variables, and to separate the direct and indirect effects of the predictors included in the model. Finally, while Random Forest accepts categorical predictors (for example, ecosystem types), this is not the case for SEM, that require certain linearity and directionality in the predictors.

## Additional information

**Accession codes:** The raw sequence data have been deposited in the GenBank SRAdatabase (BioProject accession no. PRJNA305671 for Drylands and PRJNA305672 for Scotland).

**How to cite this article:** Delgado-Baquerizo, M. *et al*. Microbial diversity drives multifunctionality in terrestrial ecosystems. *Nat. Commun.* 7:10541 doi: 10.1038/ncomms10541 (2016).

## Supplementary Material

Supplementary Information Supplementary Figures 1-15, Supplementary Table 1 and Supplementary References.

## References

[b1] Ehrlich P. R. & Ehrlich A. H. Extinction: The Causes and Consequences of the Disappearance of Species Random House (1981).

[b2] Tilman D., Lehman D. & Thompson K. Plant diversity and ecosystem productivity: theoretical considerations. Proc. Natl Acad. Sci. USA 94, 1857–1861 (1997). 11038606 10.1073/pnas.94.5.1857 PMC20007

[b3] Cardinale B. J. . The functional role of producer diversity in ecosystems. Am. J. Bot. 98, 572–592 (2011). 21613148 10.3732/ajb.1000364

[b4] Lefcheck J. S. . Biodiversity enhances ecosystem multifunctionality across trophic levels and habitats. Nat. Commun. 6, 6936 (2015). 25907115 10.1038/ncomms7936 PMC4423209

[b5] Bell T. . The contribution of species richness and composition to bacterial services. Nature 436, 1157–1160 (2005). 16121181 10.1038/nature03891

[b6] Peter H. . Function-specific response to depletion of microbial diversity. ISME J. 5, 351–361 (2011). 20686511 10.1038/ismej.2010.119 PMC3105700

[b7] van der Heijden M. G. A. . The unseen majority: soil microbes as drivers of plant diversity and productivity in terrestrial ecosystems. Ecol. Lett. 11, 296–310 (2008). 18047587 10.1111/j.1461-0248.2007.01139.x

[b8] Wagg C. . Soil biodiversity and soil community composition determine ecosystem multifunctionality. Proc. Natl Acad. Sci. USA 111, 5266–5270 (2014). 24639507 10.1073/pnas.1320054111 PMC3986181

[b9] Bardgett R. D. & van der Putten W. H. Belowground biodiversity and ecosystem functioning. Nature 515, 505–511 (2014). 25428498 10.1038/nature13855

[b10] Bodelier P. L. E. Toward understanding, managing, and protecting microbial ecosystems. Front. Microbiol. 2, 80 (2011). 21747797 10.3389/fmicb.2011.00080 PMC3128941

[b11] Miki T. . Biodiversity and multifunctionality in a microbial community: a novel theoretical approach to quantify functional redundancy. Proc. R. Soc. Lond. B 281, 20132498 (2014). 10.1098/rspb.2013.2498 PMC3871314 24352945

[b12] van der Heijden M. G. A. . Mycorrhizal fungal diversity determines plant biodiversity, ecosystem variability and productivity. Nature 396, 72 (1998).9817201

[b13] Bonkowski M. & Roy J. Soil microbial diversity and soil functioning affect competition among grasses in experimental microcosms. Oecologia 143, 232–240 (2005). 15703913 10.1007/s00442-004-1790-1

[b14] Wall D. H. . Biodiversity in the dark. Nat. Geosci. 3, 297–298 (2010).

[b15] World Bank. World Development Report, Agriculture for Development World Bank (2008).

[b16] Hooper D. U. . Interactions between above- and belowground biodiversity in terrestrial ecosystems: patterns, mechanisms, and feedbacks. BioScience 50, 1049–1061 (2000).

[b17] Wardle D. A. . Ecological linkages between aboveground and belowground biota. Science 304, 1629–1633 (2004). 15192218 10.1126/science.1094875

[b18] Jing X. . The links between ecosystem multifunctionality and above- and belowground biodiversity are mediated by climate. Nat. Commun. 2, 8159 (2015). 26328906 10.1038/ncomms9159 PMC4569729

[b19] Bardgett R. D. . Biological Diversity and Function in Soils Cambridge University Press (2005).

[b20] Mora C. . Alternative hypotheses to explain why biodiversity-ecosystem functioning relationships are concave-up in some natural ecosystems but concave-down in manipulative experiments. Sci. Rep. 4, 5427 (2014). 24962477 10.1038/srep05427 PMC4069688

[b21] Reich P. B. . Impacts of biodiversity loss escalate through time as redundancy fades. Science 336, 589–592 (2012). 22556253 10.1126/science.1217909

[b22] Gans J. . Computational improvements reveal great bacterial diversity and high metal toxicity in soil. Science 309, 1387–1390 (2005). 16123304 10.1126/science.1112665

[b23] Tedersoo L. . Fungal biogeography. Global diversity and geography of soil fungi. Science 346, 1078–1087 (2014). 10.1126/science.1256688 25430773

[b24] Maestre F. T. . Increasing aridity reduces soil microbial diversity and abundance in global drylands. Proc. Natl Acad. Sci. USA 112, 15684–15689 (2015). 26647180 10.1073/pnas.1516684112 PMC4697385

[b25] Maestre F. T. . Plant species richness and ecosystem multifunctionality in global drylands. Science 335, 214–218 (2012). 22246775 10.1126/science.1215442 PMC3558739

[b26] Yao H. . Multi-factorial drivers of ammonia oxidizer communities: evidence from a national soil survey. Environ. Microbiol. 15, 2545–2556 (2014). 23635043 10.1111/1462-2920.12141

[b27] Haegeman B. . Robust estimation of microbial diversity in theory and in practice. ISME J. 7, 1092–1101 (2013). 23407313 10.1038/ismej.2013.10 PMC3660670

[b28] Dormann C. F. . Methods to account for spatial autocorrelation in the analysis of species distributional data: a review. Ecography 30, 609–628 (2007).

[b29] van Elsas J. D. . Microbial diversity determines the invasion of soil by a bacterial pathogen. Proc. Natl Acad. Sci. USA 24, 1159–1164 (2012). 22232669 10.1073/pnas.1109326109 PMC3268289

[b30] Philippot L. . Loss in microbial diversity affects nitrogen cycling in soil. ISME J. 7, 1609–1619 (2013). 23466702 10.1038/ismej.2013.34 PMC3721106

[b31] Breiman L. Random forest. Mach. Learn. 45, 5 (2001).

[b32] Grace J. B. Structural Equation Modeling Natural Systems Cambridge University Press (2006).

[b33] Austin A. T. Water pulses and biogeochemical cycles in arid and semiarid ecosystems. Oecologia 141, 221–235 (2004). 14986096 10.1007/s00442-004-1519-1

[b34] Whitford W. G. Ecology of Desert Systems Academic Press (2002).

[b35] Schlesinger Biogeochemistry W. H. An Analysis of Global Change Academic Press (1996).

[b36] United Nations Environment Programme. World Atlas of Desertification UNEP, Edward Arnold (1992).

[b37] Hijmans R. J. . Very high resolution interpolated climate surfaces for global land areas. Int. J. Climatol. 25, 1965–1978 (2005).

[b38] Herlemann D. P. . Transitions in bacterial communities along the 2000 km salinity gradient of the Baltic Sea. ISME J. 5, 1571–1579 (2011). 21472016 10.1038/ismej.2011.41 PMC3176514

[b39] Ihrmark K. New primers to amplify the fungal ITS2 region—evaluation by 454-sequencing of artificial and natural communities. FEMS Microbiol. Ecol. 82, 666–677 (2012). 22738186 10.1111/j.1574-6941.2012.01437.x

[b40] Caporaso J. G. . QIIME allows analysis of high-throughput community sequencing data. Nat. Methods 7, 335–336 (2010). 20383131 10.1038/nmeth.f.303 PMC3156573

[b41] Magoc T. & Salzberg S. L. FLASH: fast length adjustment of short reads to improve genome assemblies. Bioinformatics 27, 2957 (2011). 21903629 10.1093/bioinformatics/btr507 PMC3198573

[b42] Aronesty E. *ea-utils*: ‘Command-line tools for processing biological sequencing data'. Available at: http://code.google.com/p/ea-utils (2011).

[b43] Edgar R. C. . UCHIME improves sensitivity and speed of chimera detection. Bioinformatics 15, 2194–2200 (2011). 21700674 10.1093/bioinformatics/btr381 PMC3150044

[b44] Cole J. R. . The Ribosomal Database Project (RDP-II): sequences and tools for high-throughput rRNA analysis. Nucleic Acids Res. 33, 294–296 (2005). 10.1093/nar/gki038 PMC539992 15608200

[b45] DeSantis T. Z. . Greengenes, a chimera-checked 16S rRNA gene database and workbench compatible with ARB. Appl. Environ. Microbiol. 72, 5069–5072 (2006). 16820507 10.1128/AEM.03006-05 PMC1489311

[b46] McDonald D. . An improved Greengenes taxonomy with explicit ranks for ecological and evolutionary analyses of bacteria and archaea. ISME J. 6, 610–618 (2012). 22134646 10.1038/ismej.2011.139 PMC3280142

[b47] Altschul S. F. . Basic local alignment search tool. J. Mol. Biol. 215, 403 (1990). 2231712 10.1016/S0022-2836(05)80360-2

[b48] Bragg L. . Fast, accurate error-correction of amplicon pyrosequences using Acacia. Nat. Methods 9, 425–426 (2012). 22543370 10.1038/nmeth.1990

[b49] Hooper D. U. . Effects of biodiversity on ecosystem functioning: a consensus of current knowledge. Ecol. Monogr. 75, 3–35 (2005).

[b50] Fierer N. . Reconstructing the microbial diversity and function of pre-agricultural tallgrass Prairie soils in the United States. Science 342, 621–624 (2013). 24179225 10.1126/science.1243768

[b51] Pérez-Valera E. . Phylogenetic structure of soil bacterial communities predicts ecosystem functioning. FEMS Microbiol. Ecol. 91, pii: fiv031 doi:10.1093/femsec/fiv031 (2015). 25873469

[b52] Price M. N. . FastTree 2-approximately maximum-likelihood trees for large alignments. Plos ONE 5, 3 (2010). 10.1371/journal.pone.0009490 PMC2835736 20224823

[b53] Faith D. P. & Baker A. M. Phylogenetic diversity (PD) and biodiversity conservation: some bioinformatics challenges. Evol. Bioinform. 2, 121–128 (2006). PMC2674678 19455206

[b54] Jax K. Ecosystem Functioning Cambridge University Press (2010).

[b55] Johnson S. L. . Increased temperature and altered summer precipitation have differential effects on biological soil crusts in a dryland ecosystem. Global Change Biol. 18, 2583–2593 (2012).

[b56] Kuske C. R. . Comparison of soil bacterial communities in rhizospheres of three plant species and the interspaces in an arid grassland. Appl. Environ. Microbiol. 68, 1854–1863 (2002). 11916705 10.1128/AEM.68.4.1854-1863.2002 PMC123825

[b57] Campbell C. D. . A rapid microtiter plate method to measure carbon dioxide evolved from carbon substrate amendments so as to determine the physiological profiles of soil microbial communities by using whole soil. Appl. Environ. Microbiol. 69, 3593–3599 (2003). 12788767 10.1128/AEM.69.6.3593-3599.2003 PMC161481

[b58] Pinchuk G. E. . Utilization of DNA as a sole source of phosphorus, carbon, and energy by Shewanella spp.: ecological and physiological implications for dissimilatory metal reduction. Appl. Environ. Microbiol. 74, 1198–1208 (2008). 18156329 10.1128/AEM.02026-07 PMC2258558

[b59] Pettorelli N. . Using the satellite-derived NDVI to assess ecological responses to environmental change. Trend Ecol. Evol. 20, 503–510 (2005). 10.1016/j.tree.2005.05.011 16701427

[b60] Allen S. E. Chemical analysis. Methods in Plant Ecology Blackwell Scientific (1986).

[b61] Tiessen H. & Moir J. O. Characterization of available P by sequential fractionation. Soil Sampling and Methods of Analysis Lewis Publishers (1993).

[b62] Paterson E. . Sequential selective dissolution of iron, aluminium, and silicon from soils. Commun. Soil Sci. Plan. 24, 2015–2023 (1993).

[b63] Byrnes J. E. K. . Investigating the relationship between biodiversity and ecosystem multifunctionality: challenges and solutions. Methods Ecol. Evol 5, 111–124 (2014).

[b64] Bradford M. . Discontinuity in the responses of ecosystem processes and multifunctionality to altered soil community composition. Proc. Natl Acad. Sci. USA 111, 14478–14483 (2014). 25246582 10.1073/pnas.1413707111 PMC4210050

[b65] Rohde K. Latitudinal gradients in species diversity: the search for the primary cause. Oikos 65, 514–527 (1992).

[b66] Wei C.-L. . Global patterns and predictions of seafloor biomass using random forest. Plos ONE 5, e15323 (2010). 21209928 10.1371/journal.pone.0015323 PMC3012679

[b67] Liaw A. & Wiener M. Classification and Regression by randomForest. R News 2/3, 18 (2002).

[b68] Fortmann-Roe S. Accurate, Adaptable, and Accessible Error Metrics for Predictive. R package version 0.9.2 (2013).

[b69] Archer E. Estimate permutation p-values for importance metrics. R package version 1.5.2 (2013).

[b70] Schermelleh-Engel K. . Evaluating the fit of structural equation models: tests of significance and descriptive goodness-of-fit measures. Methods Psychol. Res. 8, 23–74 (2003).

