# Supplementary material for: Microbial diversity drives multifunctionality in terrestrial ecosystems
Source: Nat Commun. 2016 Jan 28;7:10541. doi: 10.1038/ncomms10541 (PMC4738359; doi:10.1038/ncomms10541)
Supplement: Supplementary Information — Supplementary Figures 1-15, Supplementary Table 1 and Supplementary References. [file ncomms10541-s1.pdf]

Supplementary information

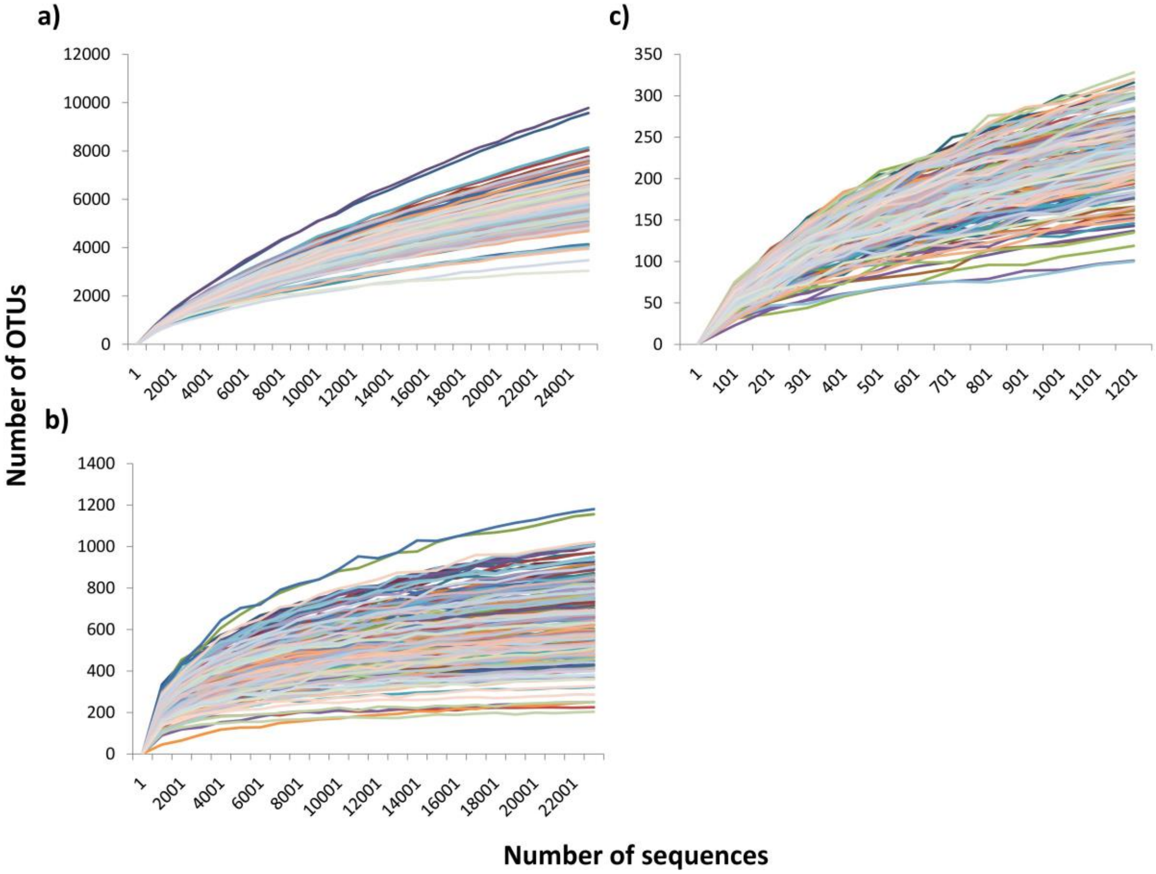

Supplementary Figure 1. Rarefaction curves for bacterial and fungal diversity in the Drylands (bacteria [a] and fungi [b]) and Scotland (bacteria [c]) datasets, respectively. Lines represent different soil samples.

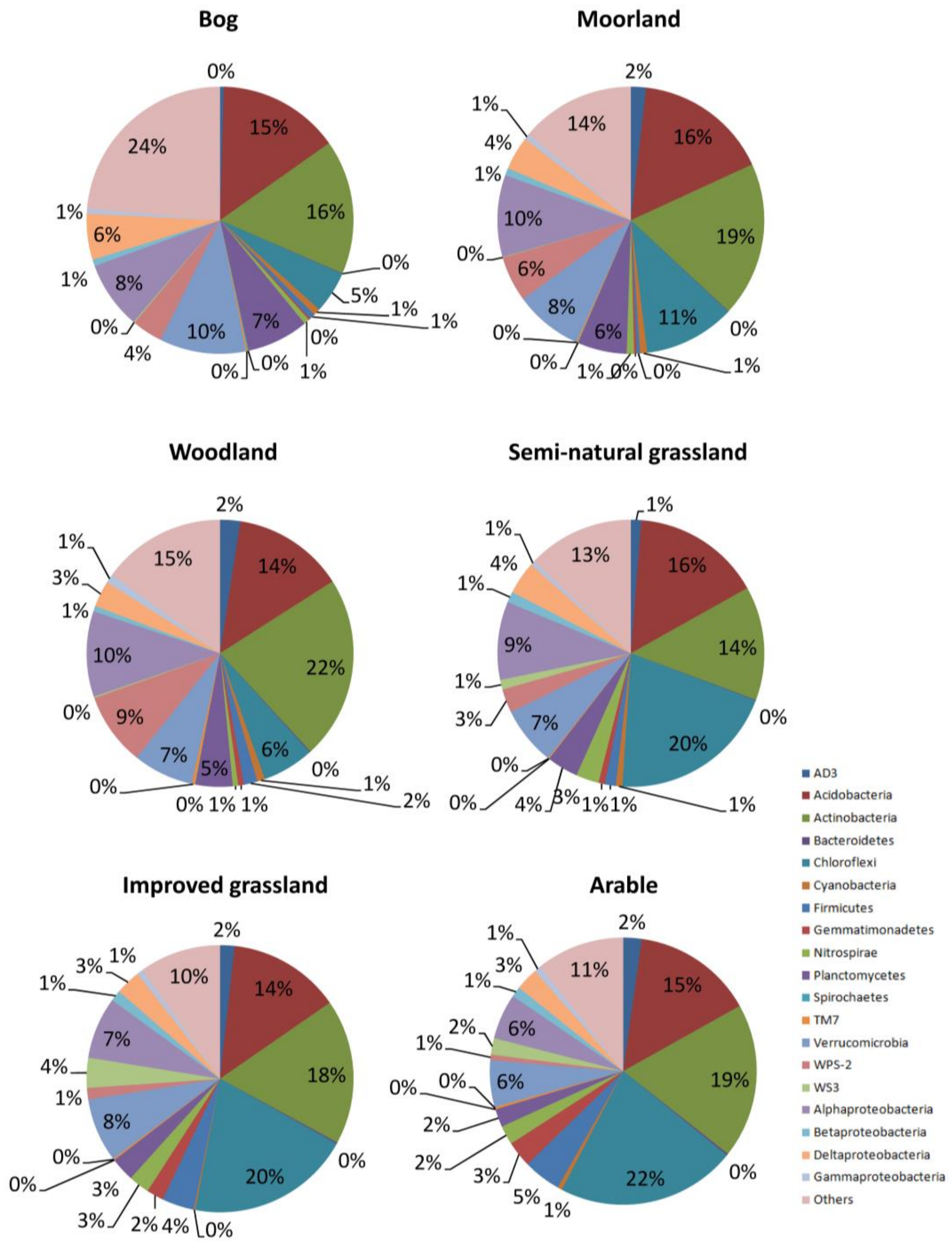

Supplementary Figure 2. Relative abundance of the main bacteria phyla/classes among different vegetation types in the Scotland dataset: arable (n = 17), improved grassland (n = 28), semi-natural grassland (n = 32), woodland (n = 29), moorland (n = 39) and bog (n = 34).

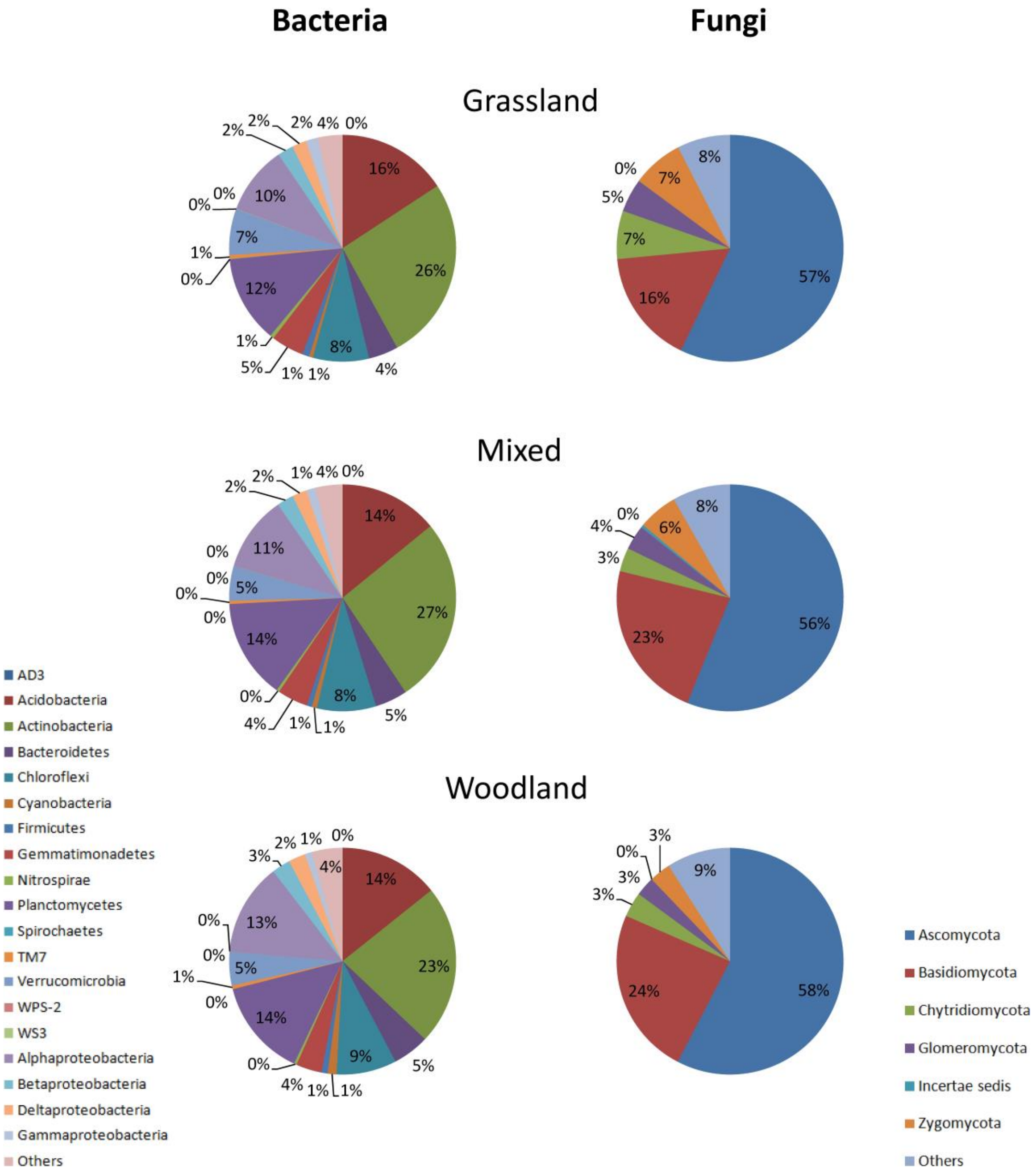

Supplementary Figure 3. Relative abundance of the main bacteria and fungi phyla/classes among different vegetation types in the Drylands dataset: grasslands (n = 17), mixed grasslands/woodlands (n = 33) and woodlands (n = 28).

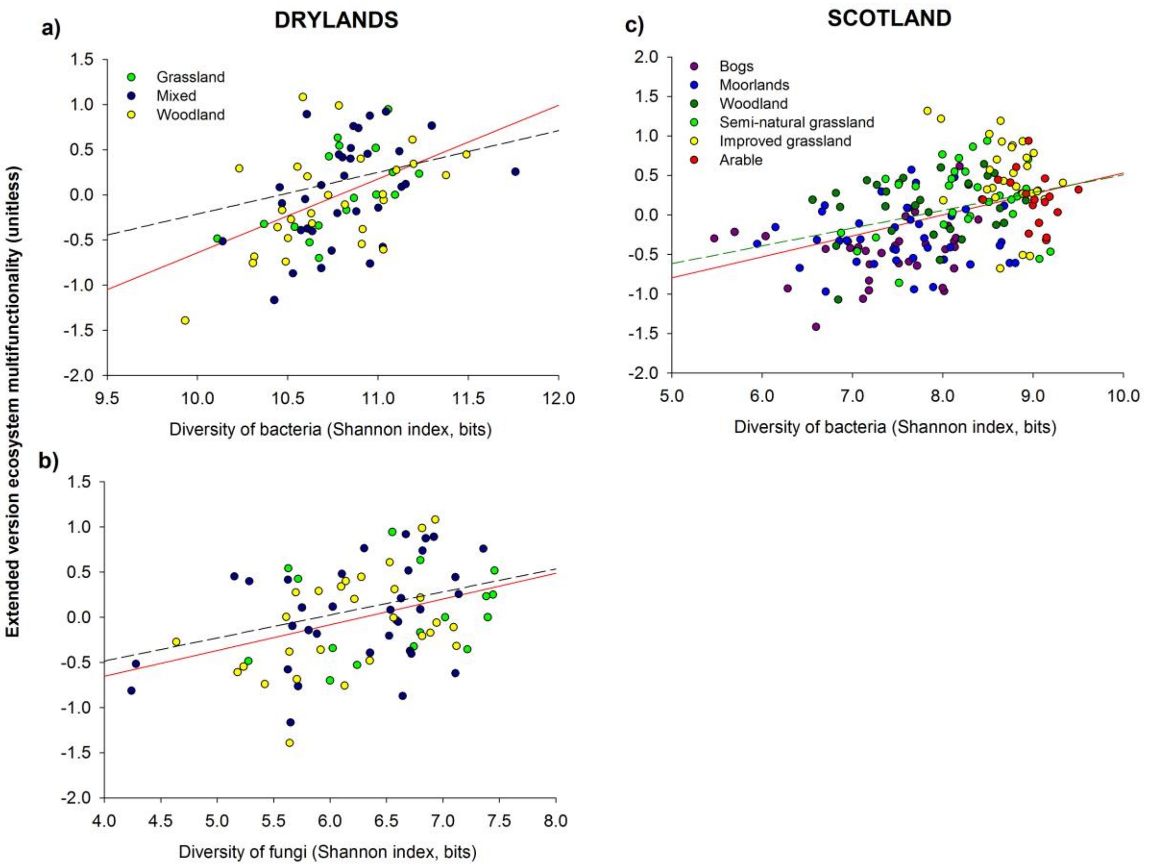

Supplementary Figure 4. Relationship between microbial diversity and ecosystem multifunctionality using 17 and eight functions available for the Drylands (bacteria [a] and fungi [b]) and Scotland (bacteria [c]) datasets, respectively. The solid and dashed lines represent the fitted ordinary least squares (OLS) and simultaneous autoregression (SAR) models, respectively. Results of regressions are as follows: (a) OLS, $R^2 = 0.234$, $P < 0.001$, AIC = 112.201; SAR, $R^2 = 0.160$, $P < 0.001$, AIC = 116.574; (b) OLS, $R^2 = 0.146$, $P < 0.001$, AIC = 120.629; SAR, $R^2 = 0.145$, $P < 0.001$, AIC = 120.776 (c) OLS, $R^2 = 0.178$, $P < 0.001$, AIC = 246.123; SAR, $R^2 = 0.174$, $P < 0.001$, AIC =247.044.

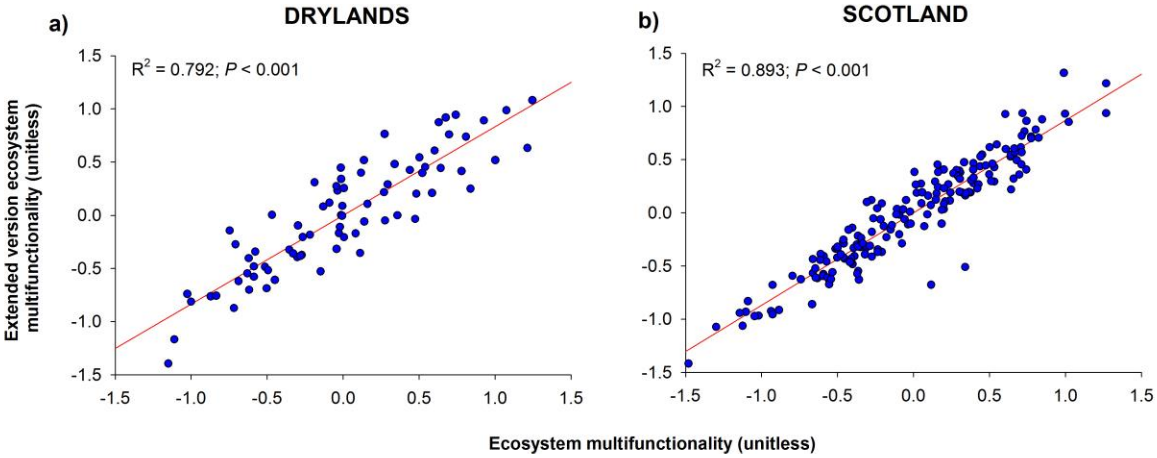

Supplementary Figure 5. Relationship between the multifunctionality index estimated with five functions (x axis) and an extended version of this index using 17 and eight functions for the Drylands (a) and Scotland (b) datasets, respectively. The solid lines represent the fitted linear regressions.

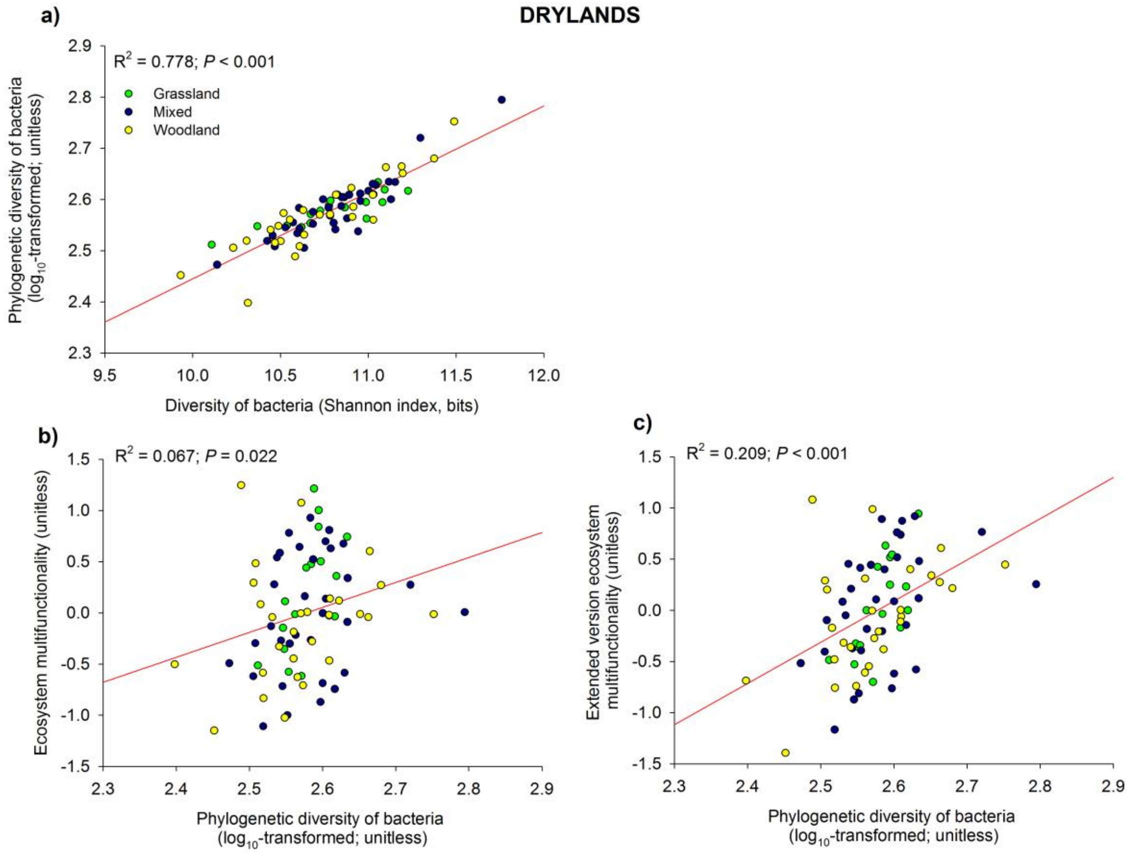

Supplementary Figure 6. Relationships between bacterial phylogenetical diversity and both Shannon diversity (a) and multifunctionality (original [b] and extended multifunctionality indices [c]) in the Drylands dataset. The solid lines represent the fitted linear regressions.

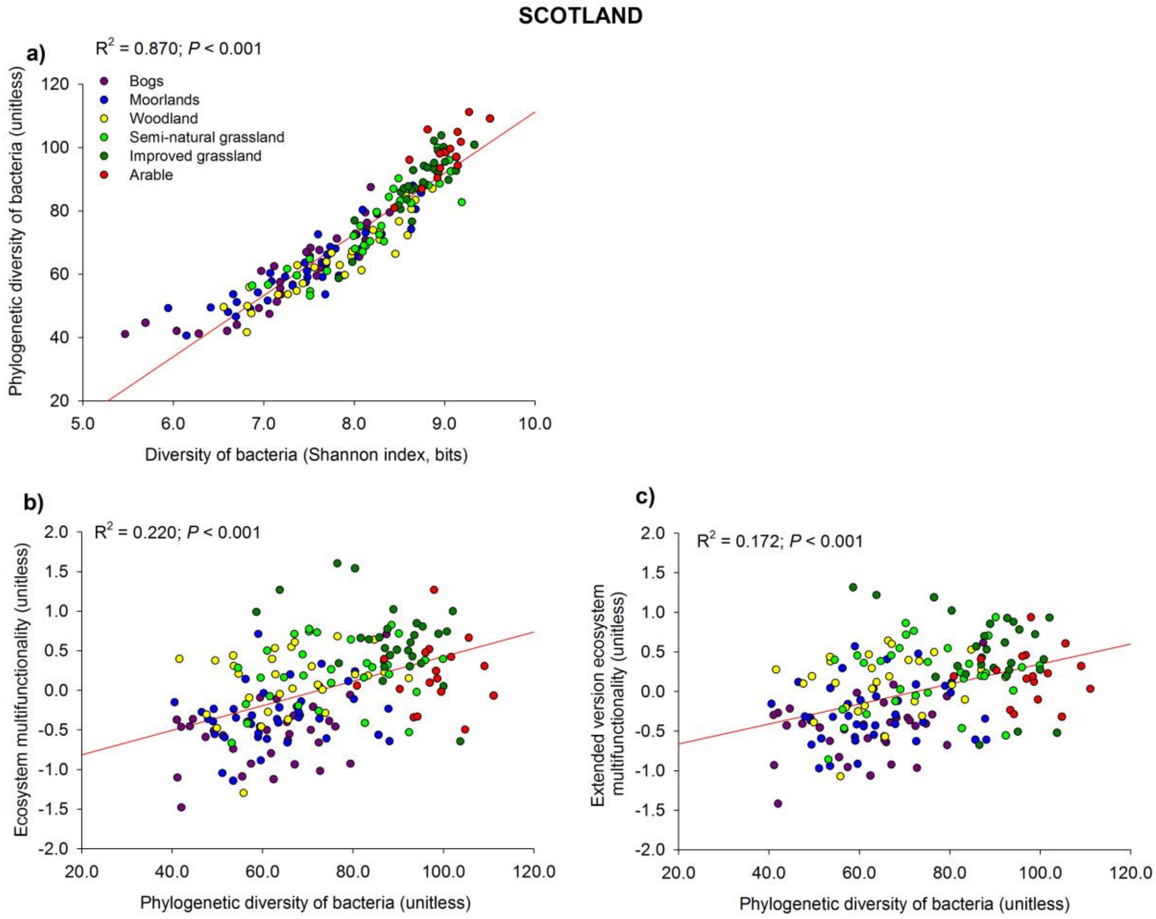

Supplementary Figure 7. Relationships between bacterial phylogenetical diversity and both Shannon diversity (a) and multifunctionality (original [b] and extended multifunctionality indices [c]) in the Scotland dataset. The solid lines represent the fitted linear regressions.

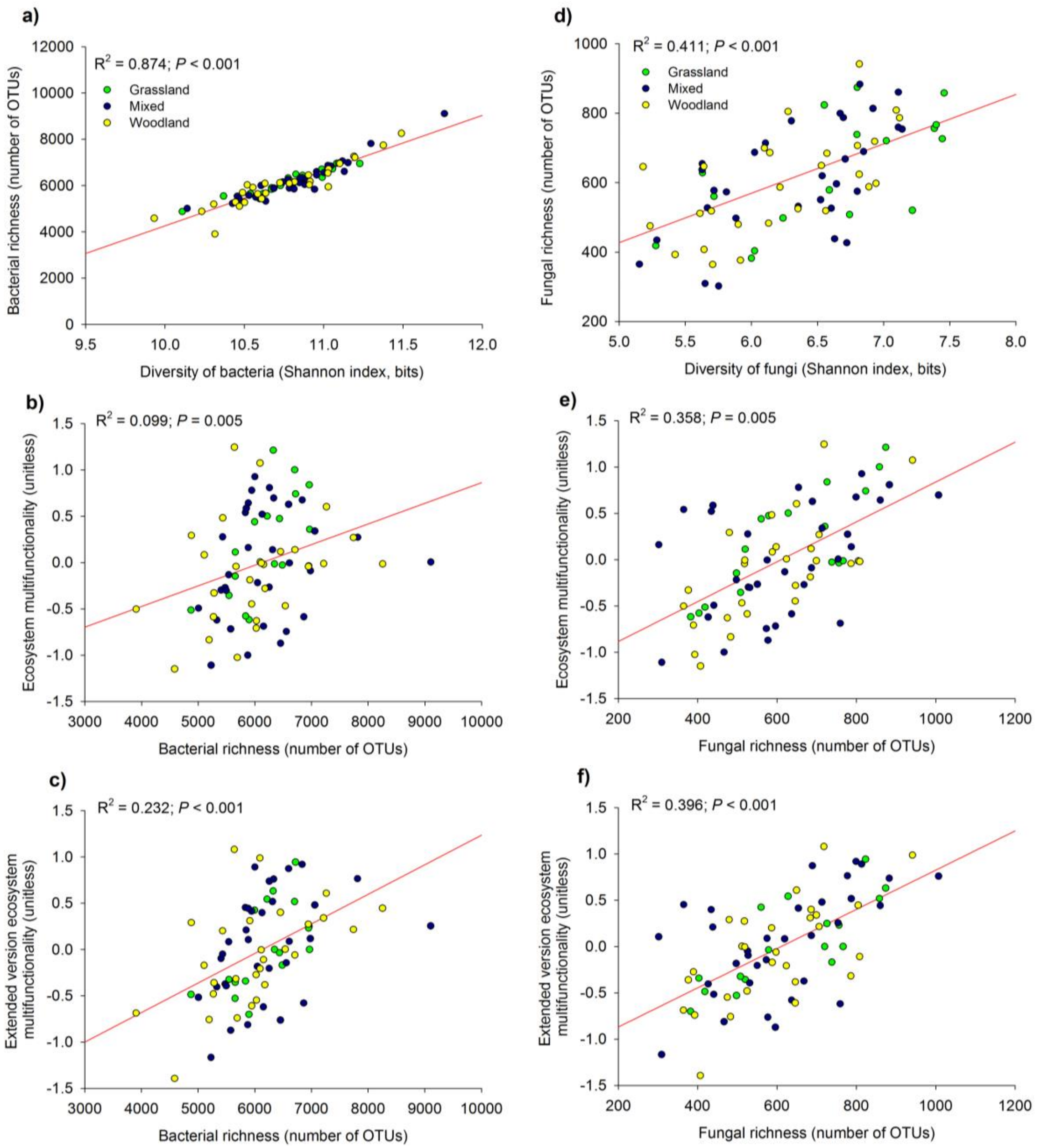

Supplementary Figure 8. Relationships between the richness of bacteria and fungi and both Shannon diversity (a and d) and multifunctionality (both original [b and e] and extended version indices [c and e]) in the Drylands dataset. The solid lines represent the fitted linear regressions.

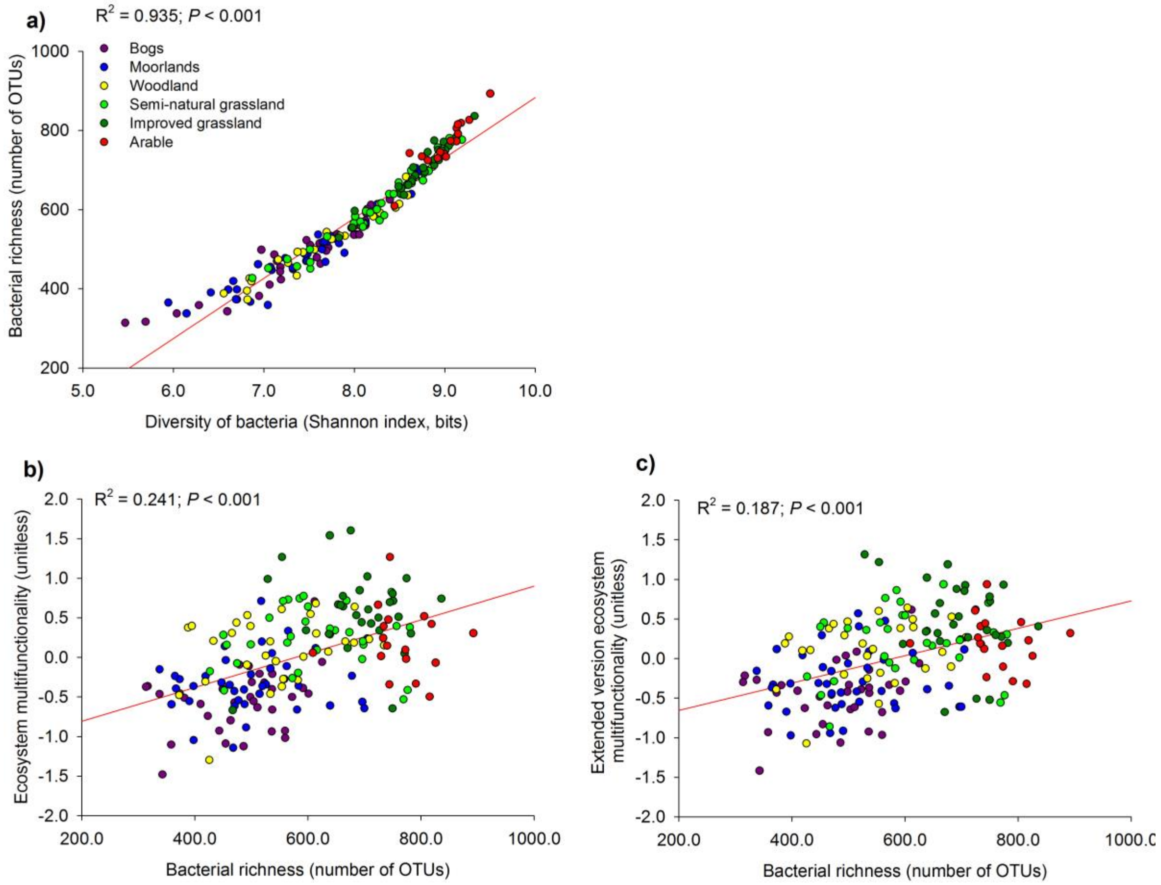

Supplementary Figure 9. Relationships between the richness of bacteria and fungi and both Shannon diversity (a and d) and multifunctionality (both original [b and e] and extended version indices [c and e]) in the Scotland dataset. The solid lines represent the fitted linear regressions.

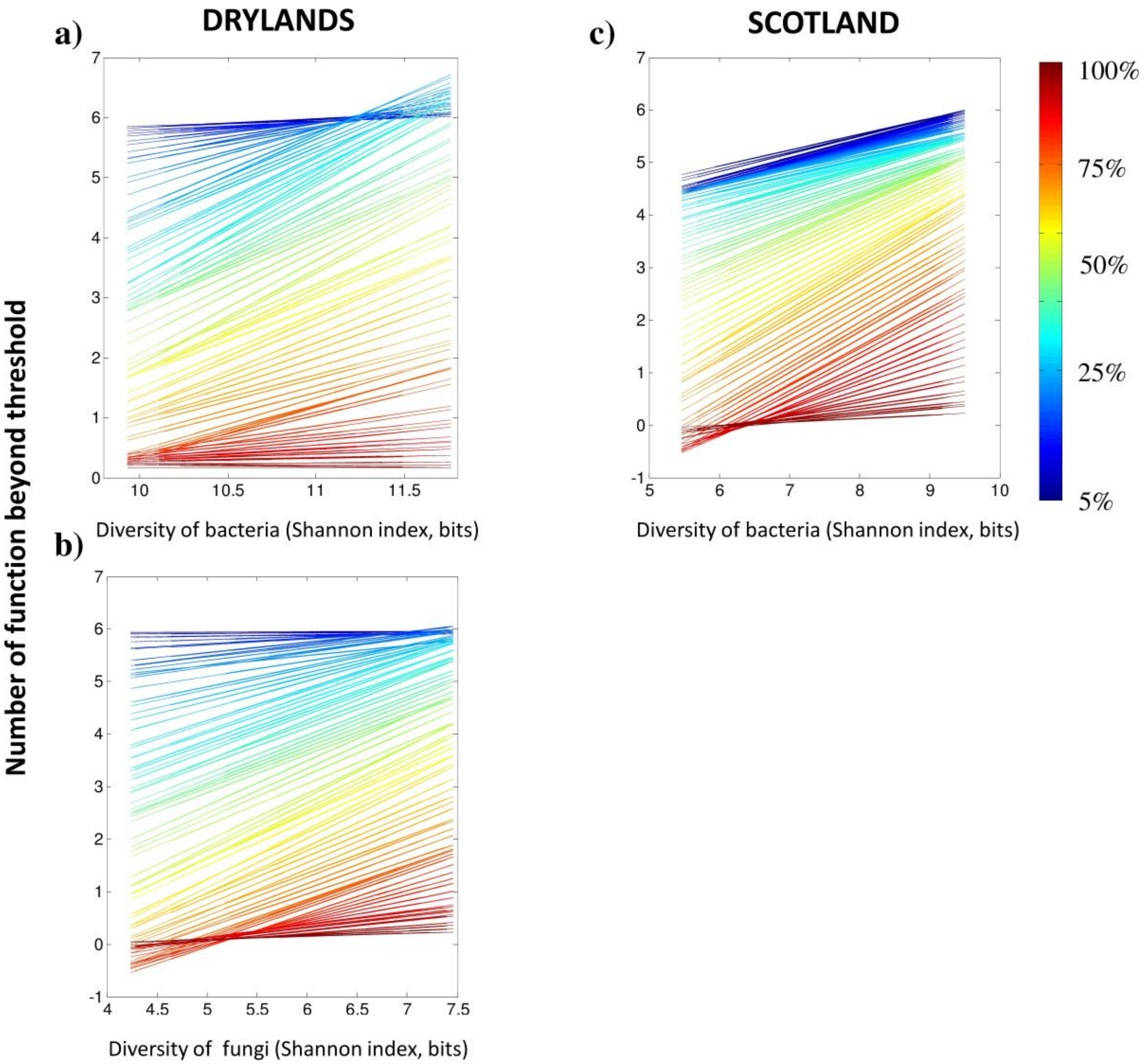

Supplementary Figure 10. Relationships between microbial diversity and the number of functions at or above a threshold (in %) of the maximum observed function for the Drylands (bacteria [a] and fungi [b]) and Scotland (bacteria [c]) datasets, respectively.

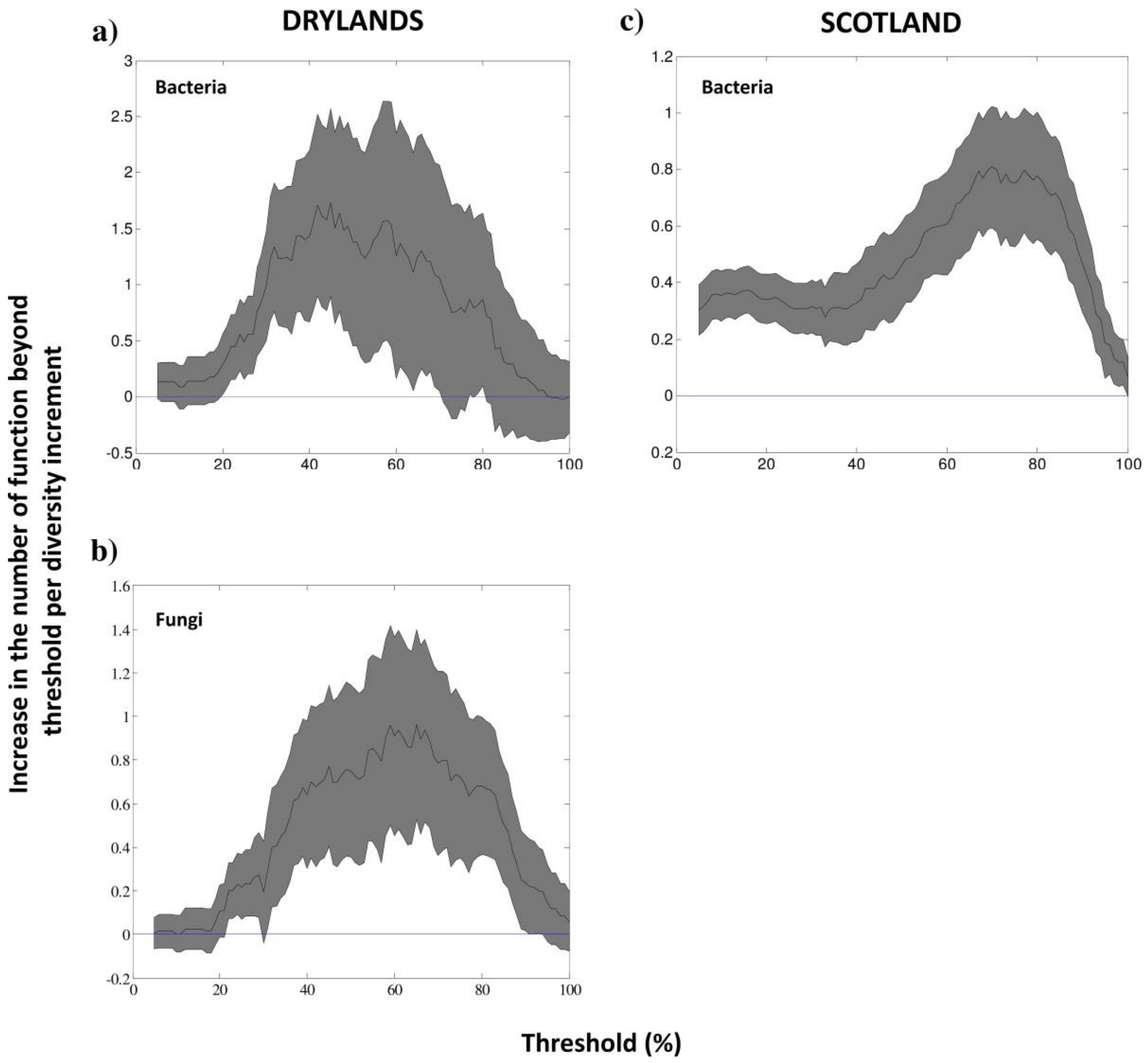

Supplementary Figure 11. Slope of the relationship between microbial diversity and the number of functions at or above a threshold (in %) of the maximum observed function for the Drylands (bacteria [a] and fungi [b]) and Scotland (bacteria [c]) datasets, respectively. Thresholds indicate the level of performance at which the role of diversity for increasing the number of functions performing beyond that level is evaluated through linear regressions. The black line and shadowed area indicate the slope and the 95% confidence interval of this regression, respectively. The threshold in which the diversity begins having a significant effect (Tmin), indicates the percentage of maximum functioning (level of performance of functions) in which the diversity can influence multifunctionality. The maximum threshold in which the effect is significant (Tmax), indicate the threshold from which diversity is not able to add functions performing

beyond that level. The value of threshold in which the effect of diversity is maximal (Tmde) and the maximum strength of the relationship (Rmde) indicates where (in which level of function performance) and how strong can be this relationship. The maximum Rmde we might find in the regressions scores theoretically 3; 1.84 and 1.5 functions per diversity increment for a b and c respectively (derived as an increase from 0 functions in minimum diversity plot to 6 functions in maximal diversity plot). Diversity is a strong driver of multifunctionality if Tmin is low; Tmax and Tmde are high and Rmde is high when compared with maximum Rmde.

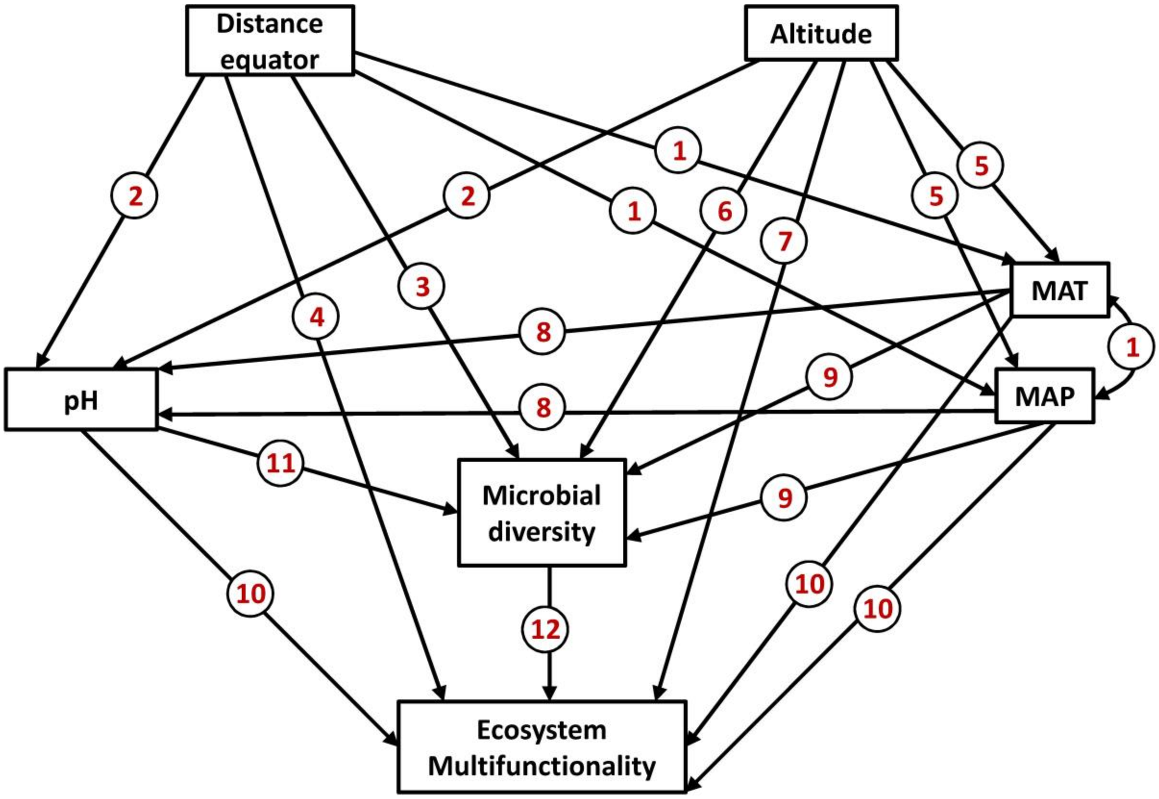

Supplementary Figure 12. *A priori* generic structural equation model (SEM) used in this study. Our model evaluated the effects of distance from the equator (absolute latitude), altitude, climate (MAT and MAP), soil pH, and microbial diversity (both bacterial and fungal diversity for Drylands and bacterial diversity for Scotland) on ecosystem multifunctionality. The numbers in the arrows denote example references used to support our predictions (see Supplementary references below).

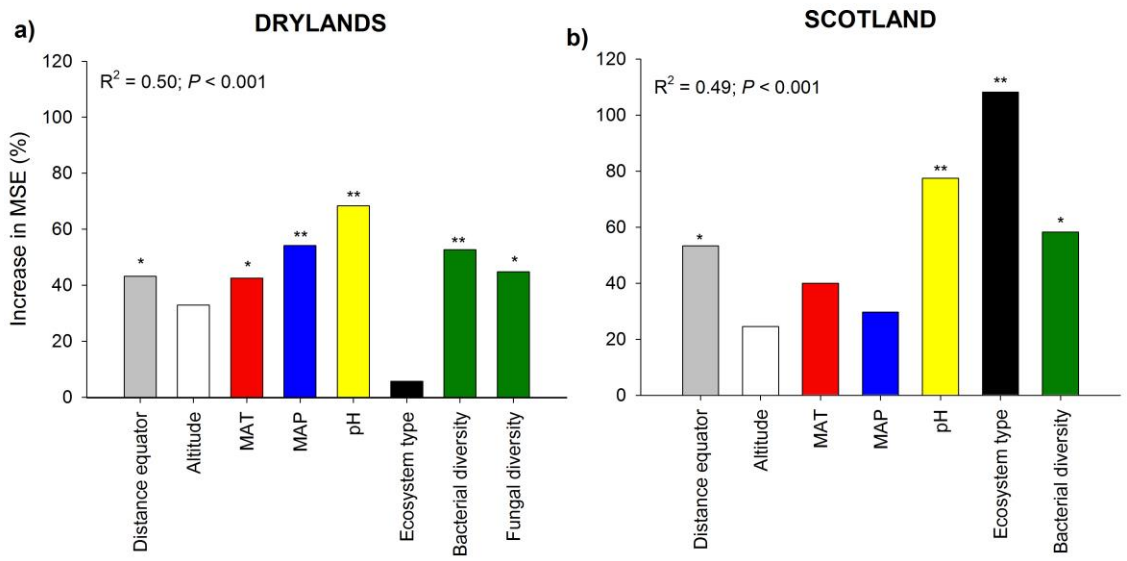

Supplementary Figure 13. Random Forest mean predictor importance (% of increase of mean square error) of environmental drivers (including vegetation type) and microbial diversity (Shannon index, bits) on ecosystem multifunctionality. Significance levels of each predictor are as follows: *p < 0.05 and **p<0.01. MAT = Mean annual temperature; MAP = mean annual precipitation.

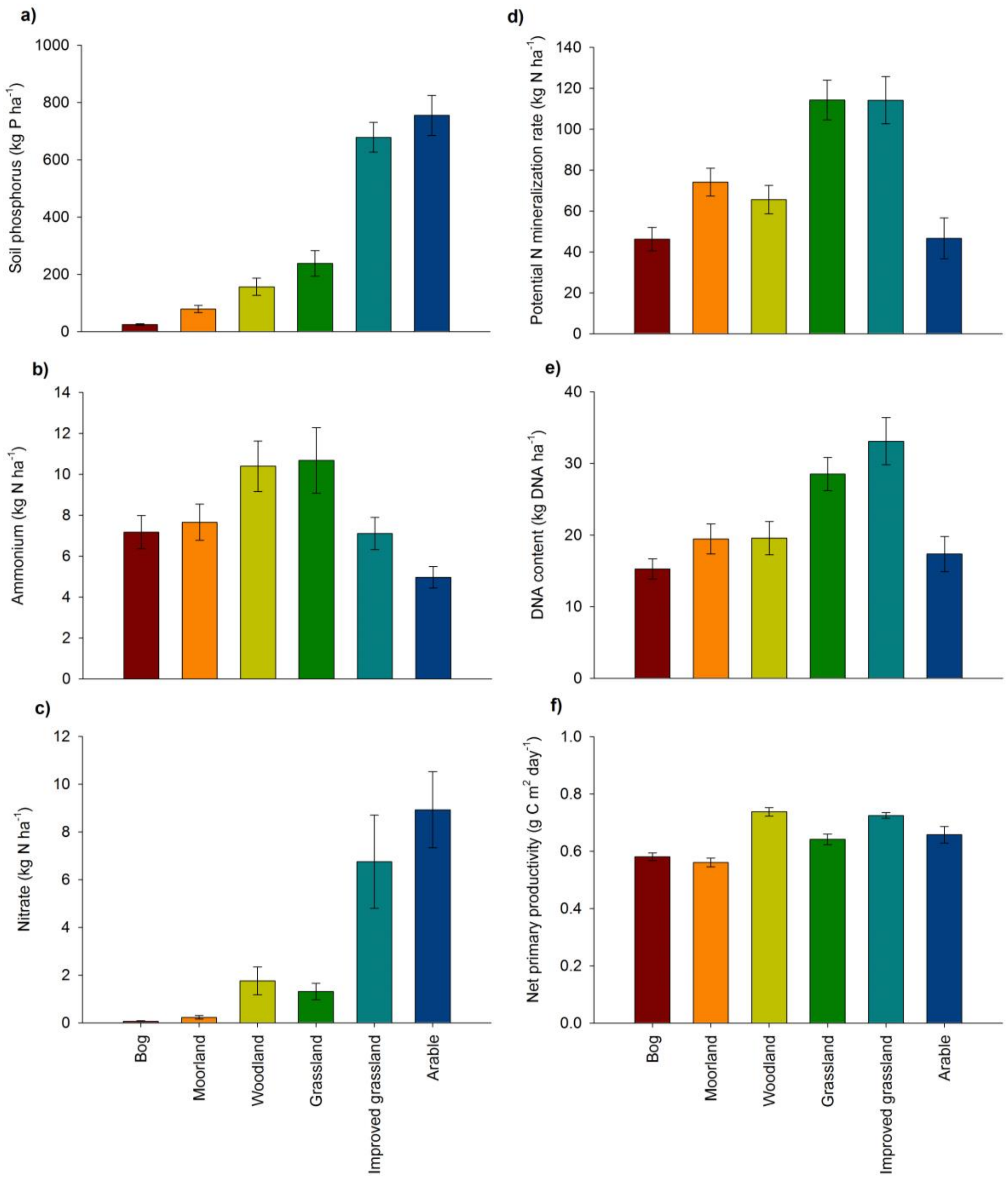

Supplementary Figure 14. Mean values for six ecosystem functions among different vegetation types in the Scotland dataset: arable (n = 17), improved grassland (n = 28), semi-natural grassland (n = 32), woodland (n = 29), moorland (n = 39) and bog (n = 34). Bar graphs represent means and SE.

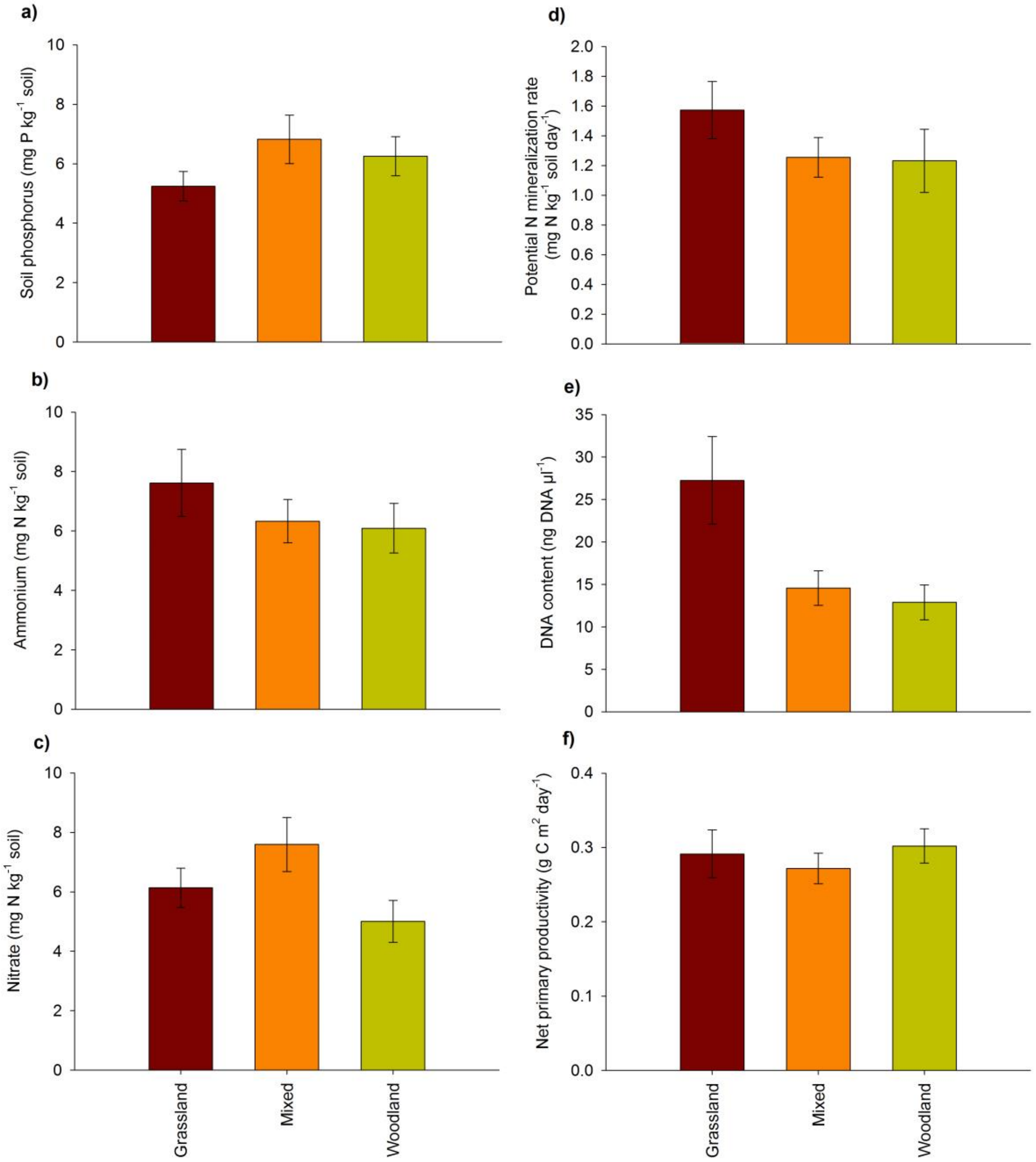

Supplementary Figure 15. Mean values for six ecosystem functions among different vegetation types in the Drylands dataset: grasslands (n = 17), mixed grasslands/woodlands (n = 33) and woodlands (n = 28). Bar graphs represent means and SE.

Supplementary Table 1. Correlation coefficients (Spearman´s ρ) between microbial diversity and both the individual functions evaluated in this study and the multifunctionality index calculated with all possible combinations of two, three, four and five functions (n = 78 and 179 for Drylands and Scotland, respectively).

		**Scotland**	**Drylands**	
**Funtions**		**Bacterial diversity**	**Bacterial diversity**	**Fungal diversity**
**One function**				
**Soil phosphorus**	ρ	0.687	-0.255	0.201
	P	<0.001	0.024	0.077
**Ammonium**	ρ	-0.182	0.448	0.422
	P	0.015	<0.001	<0.001
**Nitrate**	ρ	0.607	0.046	0.086
	P	<0.001	0.690	0.454
**Potential N mineralization rate**	ρ	0.175	0.286	0.317
	P	0.019	0.011	0.005
**DNA content**	ρ	0.277	0.376	0.428
	P	<0.001	0.001	<0.001
**Net primary productivity**	ρ	0.236	0.340	0.239
	P	0.001	0.002	0.035
**Two functions**				
**Soil phosphorus, Ammonium**	ρ	0.336	0.138	0.421
	P	<0.001	0.227	<0.001
**Soil phosphorus, Nitrate**	ρ	0.692	-0.158	0.238
	P	<0.001	0.167	0.036
**Soil phosphorus, Potential N mineralization rate**	ρ	0.529	0.046	0.366
	P	<0.001	0.687	0.001
**Soil phosphorus, DNA content**	ρ	0.559	0.100	0.432
	P	<0.001	0.384	<0.001

**Soil phosphorus, Net primary productivity**	ρ	0.515	0.056	0.322
	P	<0.001	0.624	0.004
**Ammonium, Nitrate**	ρ	0.364	0.276	0.282
	P	<0.001	0.015	0.012
**Ammonium, Potential N mineralization rate**	ρ	-0.027	0.426	0.442
	P	0.719	<0.001	<0.001
**Ammonium, DNA content**	ρ	0.003	0.496	0.509
	P	0.972	<0.001	<0.001
**Ammonium, Net primary productivity**	ρ	0.071	0.495	0.426
	P	0.342	<0.001	<0.001
**Nitrate, Potential N mineralization rate**	ρ	0.531	0.167	0.213
	P	<0.001	0.143	0.062
**Nitrate, DNA content**	ρ	0.558	0.263	0.303
	P	<0.001	0.020	0.007
**Nitrate, Net primary productivity**	ρ	0.498	0.254	0.220
	P	<0.001	0.025	0.053
**Potential N mineralization rate, DNA content**	ρ	0.251	0.408	0.461
	P	0.001	<0.001	<0.001
**Potential N mineralization rate, Net primary productivity**	ρ	0.259	0.392	0.356
	P	<0.001	<0.001	0.001
**DNA content, Net primary productivity**	ρ	0.292	0.481	0.434
	P	<0.001	<0.001	<0.001
**Three functions**				
**Soil phosphorus, Ammonium, Nitrate**	ρ	0.531	0.107	0.354
	P	<0.001	0.351	0.001
**Soil phosphorus, Ammonium, Potential N**	ρ	0.312	0.236	0.442

**mineralization rate**				
	P	<0.001	0.038	<0.001
**Soil phosphorus, Ammonium, DNA content**	ρ	0.355	0.315	0.502
	P	<0.001	0.005	<0.001
**Soil phosphorus, Ammonium, Net primary productivity**	ρ	0.341	0.294	0.436
	P	<0.001	0.009	<0.001
**Soil phosphorus, Nitrate, Potential N mineralization rate**	ρ	0.636	0.009	0.321
	P	<0.001	0.935	0.004
**Soil phosphorus, Nitrate, DNA content**	ρ	0.638	0.069	0.357
	P	<0.001	0.550	0.001
**Soil phosphorus, Nitrate, Net primary productivity**	ρ	0.611	0.065	0.314
	P	<0.001	0.574	0.005
**Soil phosphorus, Potential N mineralization rate, DNA content**	ρ	0.482	0.234	0.456
	P	<0.001	0.039	<0.001
**Soil phosphorus, Potential N mineralization rate, Net primary productivity**	ρ	0.475	0.186	0.403
	P	<0.001	0.104	<0.001
**Soil phosphorus, DNA content, Net primary productivity**	ρ	0.537	0.136	0.453
	P	<0.001	0.237	<0.001
**Ammonium, Nitrate, Potential N mineralization rate**	ρ	0.333	0.298	0.337
	P	<0.001	0.008	0.003
**Ammonium, Nitrate, DNA content**	ρ	0.400	0.377	0.394

	P	<0.001	0.001	<0.001
**Ammonium, Nitrate, Net primary productivity**	ρ	0.360	0.384	0.333
	P	<0.001	0.001	0.003
**Ammonium, Potential N mineralization rate, DNA content**	ρ	0.497	0.316	0.360
	P	<0.001	0.005	0.001
**Ammonium, Potential N mineralization rate, Net primary productivity**	ρ	0.127	0.472	0.444
	P	0.089	<0.001	<0.001
**Ammonium, DNA content, Net primary productivity**	ρ	0.157	0.543	0.497
	P	0.036	<0.001	<0.001
**Nitrate, Potential N mineralization rate, DNA content**	ρ	0.497	0.316	0.360
	P	<0.001	0.005	0.001
**Nitrate, Potential N mineralization rate, Net primary productivity**	ρ	0.488	0.306	0.314
	P	<0.001	0.006	0.005
**Nitrate, DNA content, Net primary productivity**	ρ	0.503	0.430	0.388
	P	<0.001	<0.001	<0.001
**Potential N mineralization rate, DNA content, Net primary productivity**	ρ	0.288	0.483	0.458
	P	<0.001	<0.001	<0.001
**Four functions**				
**Soil phosphorus, Ammonium, Nitrate, Potential N mineralization rate**	ρ	0.488	0.186	0.401
	P	<0.001	0.103	<0.001
**Soil phosphorus,**	ρ	0.526	0.251	0.446

**Ammonium, Nitrate, DNA content**				
	P	<0.001	0.027	<0.001
**Soil phosphorus, Ammonium, Nitrate, Net primary productivity**	ρ	0.499	0.236	0.389
	P	<0.001	0.038	<0.001
**Soil phosphorus, Ammonium, Potential N mineralization rate, DNA content**	ρ	0.327	0.345	0.507
	P	<0.001	0.002	<0.001
**Soil phosphorus, Ammonium, Potential N mineralization rate, Net primary productivity**	ρ	0.329	0.321	0.462
	P	<0.001	0.004	<0.001
**Soil phosphorus, Ammonium, DNA content, Net primary productivity**	ρ	0.377	0.395	0.512
	P	<0.001	<0.001	<0.001
**Soil phosphorus, Nitrate, Potential N mineralization rate, DNA content**	ρ	0.595	0.214	0.438
	P	<0.001	0.060	<0.001
**Soil phosphorus, Nitrate, Potential N mineralization rate, Net primary productivity**	ρ	0.595	0.144	0.373
	P	<0.001	0.209	0.001
**Soil phosphorus, Nitrate, DNA content, Net primary productivity**	ρ	0.595	0.214	0.438
	P	<0.001	0.060	<0.001
**Soil phosphorus, Potential N mineralization rate, DNA content, Net primary**	ρ	0.474	0.317	0.480

**productivity**				
	P	<0.001	0.005	<0.001
**Ammonium, Nitrate, Potential N mineralization rate, DNA content**	ρ	0.353	0.379	0.419
	P	<0.001	0.001	<0.001
**Ammonium, Nitrate, Potential N mineralization rate, Net primary productivity**	ρ	0.348	0.408	0.393
	P	<0.001	<0.001	<0.001
**Ammonium, Nitrate, DNA content, Net primary productivity**	ρ	0.401	0.473	0.438
	P	<0.001	<0.001	<0.001
**Ammonium, Potential N mineralization rate, DNA content, Net primary productivity**	ρ	0.166	0.522	0.503
	P	0.026	<0.001	<0.001
**Nitrate, Potential N mineralization rate, DNA content, Net primary productivity**	ρ	0.476	0.427	0.408
	P	<0.001	<0.001	<0.001
**Soil phosphorus, Ammonium, Nitrate, Potential N mineralization rate**	ρ	0.488	0.186	0.401
	P	<0.001	0.103	<0.001
**Soil phosphorus, Ammonium, Nitrate, DNA content**	ρ	0.526	0.251	0.446
	P	<0.001	0.027	<0.001
**Soil phosphorus, Ammonium, Nitrate, Net primary productivity**	ρ	0.499	0.236	0.389

	P	<0.001	0.038	<0.001
**Ammonium, Nitrate, Potential N mineralization rate, DNA content**	ρ	0.353	0.379	0.419
	P	<0.001	0.001	<0.001
**Ammonium, Nitrate, Potential N mineralization rate, Net primary productivity**	ρ	0.348	0.408	0.393
	P	<0.001	<0.001	<0.001
**Nitrate, Potential N mineralization rate, DNA content, Net primary productivity**	ρ	0.476	0.427	0.408
	P	<0.001	<0.001	<0.001
**Five functions**				
**Soil phosphorus, Ammonium, Nitrate, Potential N mineralization rate, DNA content**	ρ	0.479	0.304	0.470
	P	<0.001	0.007	<0.001
**Soil phosphorus, Ammonium, Nitrate, Potential N mineralization rate, Net primary productivity**	ρ	0.473	0.261	0.429
	P	<0.001	0.021	<0.001
**Ammonium, Nitrate, Potential N mineralization rate, DNA content, Net primary productivity**	ρ	0.364	0.457	0.447
	P	<0.001	<0.001	<0.001
**Soil phosphorus, Ammonium, Potential N mineralization rate, DNA content, Net primary productivity**	ρ	0.341	0.387	0.500

	P	<0.001	<0.001	<0.001
**Soil phosphorus, Ammonium, Nitrate, DNA content, Net primary productivity**	ρ	0.485	0.317	0.464
	P	<0.001	0.005	<0.001
**Soil phosphorus, Nitrate, Potential N mineralization rate, DNA content, Net primary productivity**	ρ	0.613	0.197	0.453
	P	<0.001	0.084	<0.001

Supplementary references:

1. R.J. Hijmans *et al.* Very high resolution interpolated climate surfaces for global land areas. *Int. J. of Climatol.* **25**, 1965-1978 (2005).

2. P. Goovaerts. Geostatistical tools for characterizing the spatial variability of microbiological and physico-chemical soil properties. *Biol. Fertil. Soils* **27,** 315–334 (1998).

3. J. Green, & B. J. M. Bohannan. Spatial scaling of microbial biodiversity. *Trends Ecol. Evol.* **21,** 501–507 (2006).

4. M. Begon, Townsend C.R. & Harper J.L. *Ecology From Individuals to Ecosystems* (Blackwell Publishing, MA, USA, ed.4, 2006).

5. T. Kohler, & D. Maselli. Mountains and Climate Change: From Understanding to Action (Geographica Bernensia and SDC, Bern, Switzerland, 2012).

6. N.A. Lyngwi *et al.* Cultivable bacterial diversity along the altitudinal zonation and vegetation range of tropical Eastern Himalaya. *Rev Biol Trop.* 61, 467-90 (2013).

7. J.W. MacArthur. *Ecology and Evolution of Communities* (Belknap, Cambridge, MA. 1975).

8. W.H. Schlesinger. Biogeochemistry, an analysis of global change (Academic Press, San Diego, CA, USA, 1996).

9. L. Tedersoo *et al.* Fungal biogeography. Global diversity and geography of soil fungi. Science **28**, 346. doi: 10.1126/science.1256688 (2014).

10. F.T. Maestre *et al.* Plant Species Richness and Ecosystem Multifunctionality in Global Drylands. *Science* **335**, 214 (2012).

11. C.L. Lauber *et al.* Pyrosequencing-based assessment of soil pH as a predictor of soil bacterial community. *Appl. Environ. Microbiol.* 75, 5111-20 (2009).

12. X. Jing *et al.* The links between ecosystem multifunctionality and above- and belowground biodiversity are mediated by climate. *Nature Commun.* **2**, 8159. doi: 10.1038/ncomms9159 (2015).
